# Structure Architecting for Salt‐Rejecting Solar Interfacial Desalination to Achieve High‐Performance Evaporation With In Situ Energy Generation

**DOI:** 10.1002/advs.201903478

**Published:** 2020-03-31

**Authors:** Yaoxin Zhang, Ting Xiong, Dilip Krishna Nandakumar, Swee Ching Tan

**Affiliations:** ^1^ Department of Materials Science and Engineering National University of Singapore 9 Engineering drive 1 Singapore 117574 Singapore

**Keywords:** desalination, energy generation, salt‐rejecting structures, solar interfacial evaporation, structure architecting

## Abstract

The past few years have witnessed a rapid development of solar‐driven interfacial evaporation, a promising technology for low‐cost water desalination. As of today, solar‐to‐steam conversion efficiencies close to 100% or even beyond the limit are becoming increasingly achievable in virtue of unique photothermal materials and structures. Herein, the cutting‐edge approaches are summarized, and their mechanisms for photothermal structure architecting are uncovered in order to achieve ultrahigh conversion efficiency. Design principles to enhance evaporation performance and currently available salt‐rejection strategies for long‐term desalination are systematically investigated. The guidelines to utilize every component in solar desalination systems for simultaneous in situ energy generation are also revealed. Finally, opportunities and challenges for future works in this field are also discussed and concluded.

## Graphical abstract

How to achieve high‐performance of photothermal conversion for efficient solar desalination is discussed in detail from a structural point of view. Strategies to enable salt‐rejection and simultaneous energy generation with tailored structures are also systematically summarized. Further attention should be devoted to the practical implementation of this low‐cost desalination technology.

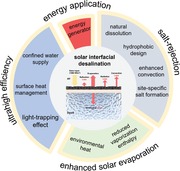

## 1 Introduction

In the face of the rapid development of modern society and ever‐growing population, the challenges in meeting the freshwater demand have increased over the past few decades.^[^ ^1^ ^]^ Desalination is developed as one of the most promising solutions to meet the challenge of the pressing global water crisis. Tremendous progress in the currently available desalination techniques have been made in the form of reduction in cost and energy consumption, especially in the reverse osmosis process.^[^ ^2^, ^3^ ^]^ However, currently, desalination plants based on the dominant membrane based technologies can only be found in service in high‐income countries and to meet essential water demand in developing countries and remote areas, the developments of alternative approaches are therefore encouraged. The need for low‐cost methods to produce clean water from seawater with minimum infrastructure and energy requirement has given rise to research thrusts being focused on the development of solar‐driven evaporation based desalination technologies.^[^ ^4^, ^5^, ^6^, ^7^ ^]^

**Figure 1:**
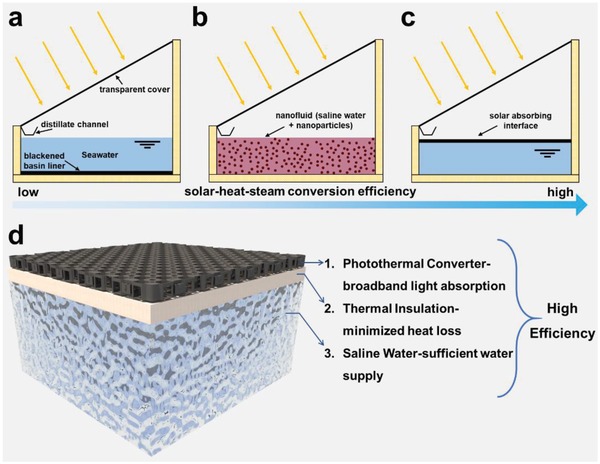
Structural comparisons of conventional solar evaporation‐based desalination configurations and solar interfacial evaporation. a) Conventional solar distiller based on bottom heating pattern with low conversion efficiency. b) Solar volumetric evaporation using nanoparticles with mediate conversion efficiency. c) Solar interfacial evaporation by heat localization with high conversion efficiency. d) Schematic introducing the key components of a solar interfacial evaporation system, and their corresponding contributions to achieving high conversion efficiency.

The research on using solar energy in the desalination process has a long history.^[^ ^3^, ^8^, ^9^ ^]^ In a traditional single‐slope basin‐like solar still (**Figure** **1**a), saline water is heated and subsequently evaporated by directly exposing to solar radiation. Solar energy is absorbed and converted into thermal energy by a black light‐absorbing liner that is situated at the bottom of the solar basin.^[^ ^10^ ^]^ Due to this design, the solar absorber is immersed in bulk seawater and it hardly receives a fraction of the incoming solar radiation owing to poor light penetration through the thick water layer. Furthermore, the produced heat is easily dissipated into the bulk water, and further lost to the surroundings through water surface radiation, convection and conduction. Due to the above reasons, the resulting solar‐to‐steam conversion efficiency is inevitably low. Nanoparticle dispersed fluid was then demonstrated to harness solar energy and found to be a great boon to direct water vapor generation.^[^ ^11^, ^12^ ^]^ A solar conversion efficiency of up to ≈70% can be possibly achieved because heat loss to bulk water is mitigated (Figure 1b).^[^ ^13^, ^14^ ^]^ However, research on this advancement didn't last because of the development of solar interfacial heating strategy, which is shown in Figure 1c. The concept of interfacial evaporation was first put forth in 2014 by two reports simultaneously,^[^ ^15^, ^16^ ^]^ and received a surge of attention and interest immediately in the following years. The most prominent feature of solar interfacial evaporation lies in the position of the solar absorber, which is at the interface between saline liquid and the above air. This special configuration not only minimizes heat loss from the solar absorber to bulk water, but also provides significantly more surface area for prompt vapor release. Also, solar radiation is photothermally localized at the surface of solar absorber, giving rise to high surface temperature, which enables fast heat transfer to water. With it, water transported from reservoir can be heated and evaporated immediately to achieve a higher rate of steam generation. In addition to the differences in heating strategies, it should be noted that all the solar stills follow the same evaporation‐condensation route for desalination and clean water collection, and to this end, a similar device structure (e.g., solar still with sloping cover) is usually employed.

There are three key components making up a solar interfacial evaporation system (Figure 1d), which are photothermal materials, heat management and water supply, and each makes hefty contribution to the final conversion efficiency.^[^ ^4^, ^17^, ^18^ ^]^ First, the optical nature of photothermal materials determines light absorption and following photothermal conversion, and in addition to that, the structural advantage of engineering a material with large surface area can further enhance steam escape. Solar absorbers with broadband light absorption are more likely to achieve extra‐high efficiency and therefore it is regarded as one of the most highly desired properties for solar evaporator materials preparation.^[^ ^19^ ^]^ Heat management in most cases refers to the engineering manipulations introduced in this system, e.g., thermal insulation, to diminish heat losses.^[^ ^20^ ^]^ The application of thermal insulation that is usually sandwiched between solar evaporator and bulk water blocks the downward heat conduction path thereby minimizing heat loss to water, leading to a more efficient photothermal conversion. Surface heat reduction is indispensable to further improve efficiency, and we have in this review given detailed discussion on this topic, which can be found in following sections. Water supply is of fundamental importance to maintain evaporation since fulfilling the maximum potential of solar evaporators requires sufficient water supply.^[^ ^21^, ^22^ ^]^ Capillary effect is usually wielded to drive water transport from reservoir to evaporation site, based on which, various strategies to transport water either in one or multiple dimensions have been proposed, and these approaches also brings other benefits, such as heat loss reduction and salt rejection.^[^ ^23^, ^24^ ^]^

**Figure 2:**
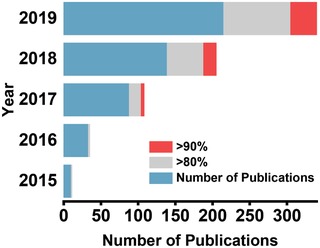
Total Number of publications on solar interfacial evaporation is given in the left diagram. Middle and right diagrams respectively the number of reports that have demonstrated solar conversion efficiency higher than 80% and 90%, respectively. The results are obtained from the Web of Science database (as on Dec 2019).

Only if a well‐balanced synergy is established between these three components, a high solar conversion efficiency can be realized.^[^ ^25^ ^]^ For this, tremendous efforts have been devoted so far which has led to extraordinary progress in this research field in a short span of time. According to the database of Web of Science, it is shown in **Figure** **2** that the number of related publications on solar interfacial evaporation is soaring up with each passing year,^[^ ^15^ ^]^ indicating the rapid development of research in this area. Further analysis by screening the performance of solar interfacial evaporators under 1 sun light intensity (1000 W m^−2^) suggests that the photothermal conversion efficiency has been improved greatly, especially in 2018, a giant leap in publication number was achieved in comparison to previous years. One sun conversion efficiency higher than 80% and 90% is nowadays becoming more and more likely. Such achievement, to our knowledge, is not merely ascribed to the advances obtained in material science, but also to the radical modifications done on the photothermal structural design, which is also of immense importance.^[^ ^26^, ^27^, ^28^ ^]^

As such, this review first gives a whole picture of the strategies that have been well‐established in recent years for achieving ultrahigh conversion efficiency in solar interfacial evaporation systems. It is also worth noting that photothermal structures that perform beyond the upper limit of photothermal conversion or at extraordinarily high evaporation rate are of huge research interest and practical value, hence, in this review, we take the opportunity to outline possible approaches that have been reported lately. Further, due to the consideration that long‐term seawater desalination may be interrupted by salt clogging issue, this review particularly provides a comprehensive summarization of currently available salt‐rejection solutions as well as advices for self‐regenerating photothermal structure construction. Last but not the least, we have further analyzed the ideas for simultaneous in situ energy generation by making use of every component in solar desalination systems. Although high‐performance solar evaporators have been widely demonstrated, we wish to point out that the practical implementation of solar desalination on a commercial scale might not produce the same results as compared to laboratory tests, since factors that would probably influence onsite performance, such as water droplet formation on the transparent condensation cover have not yet been thoroughly investigated at the current stage. These factors which affect the efficiency, if the system is implemented on a large scale deserves further analyses and contingency plans.

## 2 Design Principles to Approach 100% Efficiency

As depicted above, conversion efficiency of solar evaporation is the product of three factors, namely the solar absorber, heat management and water supply. It needs to be emphasized that they are tightly correlated to each other rather than being functionally independent, and changing one can very likely make a difference to the others.^[^ ^4^ ^]^ With a tailored photothermal design, it is possible to have all these factors synergistically combined.^[^ ^29^ ^]^ The goal to ameliorate each individual component is consistent, i.e., to minimize heat loss and maximize efficiency. Therefore, a comprehensive understanding of photothermal conversion by ascertaining its working principle and energy balance becomes particularly necessary. Hereby, this section first reveals the basics of a photothermal evaporation system, and then introduces three key strategies for conversion efficiency improvement: i) confined water supply; ii) structural light‐trapping effect; iii) surface heat management.

### 2.1 Photothermal Conversion

In a solar interfacial evaporation system, given the solar radiation is the sole energy source, when light beams are falling on photothermal materials, they are absorbed and subsequently converted into heat that will be confined on the material surface, a process known as solar heat localization.^[^ ^15^ ^]^ This confined heat raises the temperature of solar absorber and causes the water to boil resulting in steam generation which implies, the amount of localized heat available for thermal vaporization is considerably determined by light absorption. Therefore, broadband light absorption across the full spectrum of solar radiation in the range of 300 to 2500 nm is highly desired for a high‐performance photothermal material. Various materials with high solar absorption for solar steam generation have been fully developed in the past few years, and based on the way how the light is converted into heat to drive water evaporation, photothermal materials can be divided in three main categories, which are plasmonics, semiconductors, and carbon‐based materials.^[^ ^28^ ^]^

#### 2.1.1 Photothermal Materials

Photothermal conversion of metal‐based plasmonic materials is based on a physical resonance effect that is triggered in the context of a resonance achieved between the photon frequency and the natural oscillation frequency of electrons from the conduction band of the metal surface.^[^ ^30^, ^31^ ^]^ This process finally increases the metal surface's temperature, known as localized surface plasmon resonance (LSPR).^[^ ^32^ ^]^ The absorption of plasmonic materials can be further fine‐tuned to make a wider range of wavelength covered, via meticulously adjusting the size, orientational arrangement, shape, and structure at nanoscale.^[^ ^28^, ^33^ ^]^ Meanwhile, there is an increasing interest in the combination of plasmonic absorbers and substrate materials with sophisticated structures such as porous aluminum membrane,^[^ ^34^ ^]^ bacterial nanocellulose based biofoam,^[^ ^35^ ^]^ graphene oxide‐polyurethane sponge,^[^ ^36^ ^]^ silica gel,^[^ ^37^ ^]^ agarose aerogel,^[^ ^38^ ^]^ natural wood and microporous paper,^[^ ^39^, ^40^, ^41^ ^]^ etc., to further enhance light absorption. Besides, in the hope of lowering down the cost of materials, cheap materials like aluminum and titanium nitride nanoparticles employed as photothermal materials have also been studied.^[^ ^18^, ^34^, ^42^, ^43^ ^]^

Low‐cost and excellent photostability have prompted the investigation of semiconductors as efficient solar evaporators. By virtue of the relaxation process of the excited electron–hole pairs generated during light absorption, semiconductors transduce the absorbed light energy into thermal energy.^[^ ^44^ ^]^ The amount of heat generated in this process depends on the bandgap between valence band and conduction band. In a nutshell, to achieve the maximum heat generation, semiconductor materials with narrow bandgap would be more preferable. A typical example is Titanium. Wang et al. reported a nanoscale titanium sesquioxide (Ti_2_O_3_) semiconductor with an extremely narrow bandgap of ≈0.1 eV, which allows it to achieve a high light absorption of ≈92%.^[^ ^45^ ^]^ Notably, the reported conversion efficiency is as high as 92.1%, suggesting nearly 100% of the absorbed light has been converted into heat for steam generation. In addition to the titanium‐based photothermal materials, a wide range of semiconductor materials such as CuFeSe_2_,^[^ ^46^ ^]^ Cu_7_S_4_,^[^ ^47^ ^]^ black TiO_2_,^[^ ^48^ ^]^ Fe_3_O_4_, and MFe_2_O_4_,^[^ ^49^, ^50^ ^]^ have also been utilized for efficient photothermal conversion.

In comparison to the above plasmonic materials and semiconductors, carbon‐based materials have shown vast potential for efficient steam generation, especially for large‐scale applications, owing to their naturally high light absorption, low cost and great abundance. Carbon‐based materials generate heat in the form of lattice vibration when exposed to light, and the large amount of conjugated π bonds existing in carbon‐based materials require less energy for electron excitation, which makes it possible to almost capture the entire spectrum of solar radiation.^[^ ^51^, ^52^ ^]^

To date, a wide range of carbon materials has been investigated as high‐efficiency solar absorbers. In particular, various graphene‐based materials have been intensively studied, including graphene oxide (GO) film,^[^ ^53^ ^]^ GO aerogel,^[^ ^54^ ^]^ multifunctional porous graphene,^[^ ^55^ ^]^ bilayered biofoam,^[^ ^56^ ^]^ and a series of reduced graphene oxide (rGO) composites.^[^ ^57^, ^58^, ^59^, ^60^, ^61^, ^62^ ^]^ Other carbon materials such as carbon black, carbon nanotubes, and synthesis carbon have also been widely applied in combination with different substrates.^[^ ^63^, ^64^, ^65^, ^66^, ^67^, ^68^, ^69^ ^]^ In addition to the traditional carbon materials, an increasing trend toward the application of those natural materials for photothermal conversion has provoked a lot of attention. Natural materials like wood, mushroom, food waste, flour, cotton, daikon, bamboo, etc., after a common thermal treatment, namely carbonization, can obtain excellent light‐absorbing ability, and thus are widely employed in solar evaporation systems.^[^ ^70^, ^71^, ^72^, ^73^, ^74^, ^75^, ^76^, ^77^, ^78^, ^79^ ^]^ These low‐cost natural materials are of great abundance, which befits future large‐scale applications.

#### 2.1.2 Energy Balance

**Figure 3:**
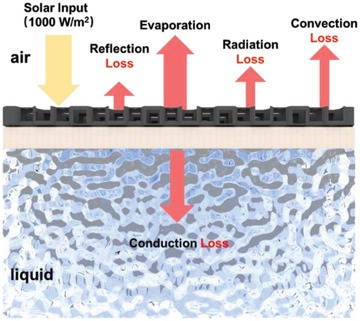
Energy balance of a solar interfacial evaporation system. Identifying the energy balance facilitates our understanding of solar evaporation, and is of benefit to making effective strategies for heat loss reduction. The reflection loss can be eliminated by using solar absorbers with high light absorption; conduction loss has been particularly reduced with the application of thermal insulation; surface heat loss including convection and radiation loss requires future exploration, and currently, several reports have demonstrated that spatial structures are able to confine and recover the surface heat loss.

Evaporation is an energy‐intensive process involving complex heat transfer and exchange. A proper understanding of the energy balance within the solar evaporation system would no doubt be of instructive significance for system optimization and performance improvement. As stated earlier, a part of the absorbed light is converted into heat by photothermal materials to drive evaporation, while the remaining is lost via reflection. Further heat losses will also occur during evaporation, which includes the conduction loss to cool bulk water, radiation loss and convection loss to the atmosphere (**Figure** **3**). Downward conduction loss to bulk water is driven by temperature gradient and water convection, and can be quantified by recording the temperature change of water reservoir over a complete solar evaporation process. However, when calculating surface radiative and convective loss, which are caused by heat transfer from solar absorbers to the environment as a result of different temperatures, the definition of the boundary condition between them is currently controversial. In previous works, room temperature was mostly selected, but some reports advocated that if solar absorbers lost heat to room, the final calculated result would break the theoretical energy balance. Instead, the results would be more reasonable and consistent with experimental observations if the temperature of the hot water vapor zone that exists closely to the evaporation surface is considered.^[^ ^25^, ^80^, ^81^, ^82^ ^]^ However, the measurement strategies and the precise boundary condition for this is not yet settled.

The solar‐to‐steam conversion efficiency can thus be defined by the proportion of the light energy that is consumed for steam production, and it can be calculated by the following equation

(1) $$\eta \;=\frac{\dot{m}h_{\text{lv}}}{C_{\text{opt}}P_{0}}$$

in which $\dot{m}$ is the evaporation rate (unit: kg m^−2^ h^−1^), *h*_lv_ refers to the total enthalpy demanded to turn liquid water into vapor, *C*_opt_ *P*_0_ is the total solar energy input ( *P*_0_= 1 kW m^−2^, *C*_opt_ is the optical concentration). Although this equation has been well‐defined previously, attention still needs to be carefully paid for accurate efficiency calculation. First, natural evaporation under dark condition should be subtracted from the experimentally obtained evaporation flux under light illumination when determining $\dot{m}$, to ensure that the evaporation rate used in calculation is contributed from light only. *h*_lv_ includes the sensible heat of water and the latent heat of liquid‐vapor phase‐change. The calculation of the sensible heat is based on a case‐by‐case basis, largely dependent on the specific heat of water and the temperature difference between solar absorbers and bulk water. It is worth mentioning that the latent heat is not constant and should be determined with the surface temperature of solar evaporators. For example, the latent heat of water phase‐change enthalpy on a 40 °C solar absorber is 2406 kJ kg^−1^, and it would be 2256 kJ kg^−1^ on a 100 °C surface. However, there are still a lot of reports using 2256 kJ kg^−1^ in the calculation, while the evaporator' temperature is far below 100 °C. Additionally, it is often overlooked that the exposure area of the entire setup should be tightly confined within the solar absorber' surface, in order to avoid any extra heat input either from solar light or the heated adjacent surroundings.

It is also of interest that, the thermal path diagram gives hint for possible photothermal structure modifications and valuable research opportunities. For example, energy stored in steam can be recycled for electricity generation and multistage steam generation,^[^ ^83^, ^84^ ^]^ and the naturally established temperature difference between solar absorber and bulk water that results in conduction loss can be utilized in thermoelectric applications.^[^ ^68^, ^79^, ^85^ ^]^

### 2.2 Confined Water Supply

**Figure 4:**
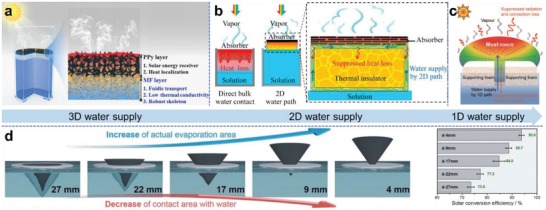
Various water supply strategies to reduce conductive heat loss. a) 3D water supply enabled by a porous melamine foam‐based solar absorber that is directly soaking in water. Reproduced with permission.^[^ ^88^ ^]^ Copyright 2019, Elsevier. b) 2D water supply from a thin water layer attached underneath the solar absorber. A thermal insulator integrated with the 2D water path design can further suppress heat loss to bulk water. Reproduced with permission.^[^ ^53^ ^]^ Copyright 2016, National Academy of Sciences. c) 1D water supply using a mushroom. Water is delivered to solar heating area through the mushroom's stipe, and as a result, the greatly reduced contact area between water and solar absorber minimizes conduction heat loss. Reproduced with permission.^[^ ^96^ ^]^ Copyright 2017, Wiley‐VCH. d) Benefits of confined water supply. It is shown that by lowering down the dimension of water supply for a cone‐shape solar evaporator, the actual evaporation area increases and the water contact area decreases, thus leading to suppressed heat loss to bulk water and higher solar conversion efficiency. Reproduced with permission.^[^ ^101^ ^]^ Copyright 2018, Royal Society of Chemistry.

Sufficient and continuous water supply is a guarantee of fulfilling maximum evaporation capability of photothermal materials, however, overly direct contact with water inevitably makes solar absorbers lose heat to the underlying water body through thermal conduction, resulting in poor conversion efficiency. The idea of confined water supply was put forward to resolve this problem, which refers to that the feed water for evaporation is transported from bulk water to solar absorbers through cramped water pathways with minimal material‐water contact. To this end, 3D, 2D, and 1D water pathways were progressively developed. The definition of the mode of water transport in a solar evaporation system is relative. It depends on the means by which the evaporator materials and bulk water are surfaced and how it receives water from capillary substrate. In the early development stage of solar interfacial evaporation, solar evaporators are directly floating on water surface without thermal insulation layer, and water is transported through interconnected and porous substrate (e.g., wood stem, melamine foam) to the top photothermally active surface, which is usually defined as 3D water pathway (**Figure** **4**a).^[^ ^16^, ^86^, ^87^, ^88^ ^]^ One major drawback of 3D water transport structures is they are mostly composed of open pores, and the thermal conductivity of the openly porous structures would increase considerably when soaked in water. Due to this reason, a capillary wicking substrate with low thermal conductivity under wet condition is highly desired to reduce conductive loss.^[^ ^89^, ^90^ ^]^

More confined water pathways were proposed to minimize the contact with water, thus saving the heat from conduction loss. 2D water supply employs hydrophilic intermediary materials such as cellulose,^[^ ^91^ ^]^ cotton and silk fabrics,^[^ ^47^, ^92^, ^93^, ^94^ ^]^ vertically oriented graphene structures,^[^ ^81^ ^]^ air‐laid paper,^[^ ^95^ ^]^ etc., for water delivery. In this way only a thin confined water layer is in contact with solar absorbers to supply water for evaporation (Figure 4b). Conduction heat loss can be further suppressed by combining a thermal insulator with the water conveyer. With this tailored 2D water pathway, Zhu and co‐workers demonstrated a 40–50% efficiency increase when compared to the direct bulk water contact design.^[^ ^53^ ^]^ Similarly, 1D water supply was put forth. Analogous to the mechanism of water transport to the pileus in a mushroom through its stipe as shown in Figure 4c,^[^ ^96^ ^]^ water can also be pumped upward to the photothermal materials through a 1D artificial stem using water wicking materials.^[^ ^50^, ^97^, ^98^, ^99^, ^100^ ^]^ In this 1D water transport, downward heat loss is perfectly minimized due to neglectable contact with water transport channels. To manifest the advantage of confined water supply for solar evaporation, Wang et al. have demonstrated a good example by using a 3D photothermal cone structure.^[^ ^101^ ^]^ It is shown in Figure 4d that by lifting the cone structure to decrease its contact area with water while increasing the actual evaporation area, the resultant efficiency increases significantly. Though the constriction of water pathways helps in achieving a higher photothermal efficiency, the limited water convention between the reservoir and the evaporator material can possibly give rise to salt accumulation especially in case of desalination–a challenge that needs to be mitigated to sustain the process.^[^ ^97^, ^101^, ^102^, ^103^ ^]^

### 2.3 Light‐Trapping Structure

Light absorption is one of the key factors determining the efficiency of solar conversion. Hence, the material choice is of great importance for solar evaporator design and fabrication. Of equal importance is its inner structure. Recently, the light‐trapping effect observed within the structure of solar absorbers have attracted increasing attention as this enhances the light absorption of these materials. This phenomenon can be defined as following: mostly in sophisticated structures at microscale and nanoscale, light beams except for those that have been absorbed at the first point of contact are reflected and scattered. This unabsorbed light is subsequently trapped by the dense anfractuous surrounding structures due to multiple internal reflections resulting in an eventual reabsorption by the medium and only a small fraction of the incident light manages to go back to atmosphere, resulting in extremely high light absorption.

To our best knowledge, there are basically two strategies developed to achieve the light‐trapping effect, which are synthetically architecting hierarchical structures and direct use of substrates that are naturally and commercially available.

**Figure 5:**
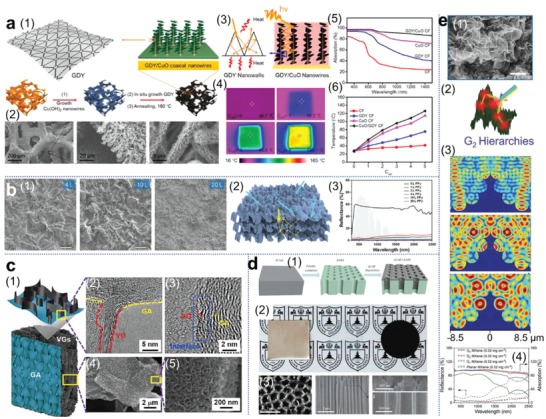
Structural design principles for enhanced light absorption by light‐trapping effect. a): 1) Schematic illustration and 2) SEM images of graphdiyne (GDY)‐based hierarchical architecture. 3) Multiple reflection induced light‐trapping effect for 4) enhanced light absorption. 5,6) The hierarchical GDY/CuO‐GF exhibiting higher surface temperature compared to other control structures due to high light absorption. Reproduced with permission.^[^ ^104^ ^]^ Copyright 2017, American Chemical Society. b): 1) SEM images of wrinkle structures obtained by controlling the number of PPy layers; 2) Light‐trapping effect in multilayer PPy wrinkle for 3) enhanced light absorption. Reproduced with permission.^[^ ^105^ ^]^ Copyright 2019, Wiley‐VCH. c): 1) Schematic of VG/GA composite. VG: vertically oriented graphene nanosheets; GA: graphene aerogel. 2,3) High‐resolution TEM images, and 4,5) SEM images of VG/GA. Reproduced with permission.^[^ ^81^ ^]^ Copyright 2019, Elsevier. d): 1) Fabrication process, 2) Optical photographs and corresponding SEM images of low‐cost hierarchical aluminum‐based solar absorber with plasmonic Al nanoparticles. Reproduced with permission.^[^ ^43^ ^]^ Copyright 2016, Springer Nature. e): 1) SEM images of a biomimetic MXene‐based crumbled structure and 2) the light‐trapping effect induced by the crumbles. 3) Simulated electric field distributions of MXene structures at wavelengths (λ) of 1500 (top), 2000 (middle), and 2500 nm (bottom) showing dense hot spots around the crumbles, indicating field confinement induced by strong localized surface plasmon resonances. 4) Light absorption of MXene crumbled texture. Reproduced with permission.^[^ ^107^ ^]^ Copyright 2019, Wiley‐VCH.

This effect has been adequately demonstrated and elucidated in the synthesis of a hierarchical graphdiyne‐based architecture by Zhang and co‐workers.^[^ ^104^ ^]^ In this example as shown in **Figure** **5**a, Cu(OH)_2_ nanowires were first grown on a porous copper foam and were covered by a layer of graphdiyne nanosheets. The final GDY/CuO‐CF composite is able to absorb 92% of the light in the range of 300–1500 nm. Optical absorption and surface temperature of GDY‐CF and CuO‐CF under solar irradiation have been proved lower than the hierarchical structure, suggesting the validation of light‐trapping effect. Xu and co‐workers coated multilayer polypyrrole (PPy) nanosheets on paper substrate and obtained wrinkle structures (Figure 5b).^[^ ^105^ ^]^ It was observed that as the number of PPy layers increases, the population and amplitude of wrinkles increase accordingly, and similar observation was also obtained in the corresponding light absorption, as a result of light‐trapping within the developed structure. It was also found that the absorption reached to its maximum (≈99%) with 10 layers of PPy nanosheets, implying that this effect is deliberately tunable. Photothermal structures with orderly and randomly aligned structures,^[^ ^56^, ^58^, ^90^, ^106^ ^]^ especially those vertically oriented nanostructures such as the graphene nanosheets highlighted in Figure 5c,^[^ ^81^ ^]^ have also been considered as an important group of materials with superior properties for solar thermal conversion. These oriented structures not only enable broadband light absorption, but also, they are further capable of ultrafast solar thermal response. Metal substrates for light trapping have also been investigated. Zhu and co‐workers fabricated a novel nanoporous aluminum membrane as shown in Figure 5d, and after decorating it with self‐assembled plasmonic aluminum nanoparticles, this low‐cost all Al structure was found to absorb 96% of a broad solar spectrum.^[^ ^43^ ^]^ In a recent report by Li et al.,^[^ ^107^ ^]^ this effect demonstrated in a MXene‐based hierarchical crumbling structure (93.2% light absorption) was theoretically validated by simulated electric field distributions (Figure 5e).

**Figure 6:**
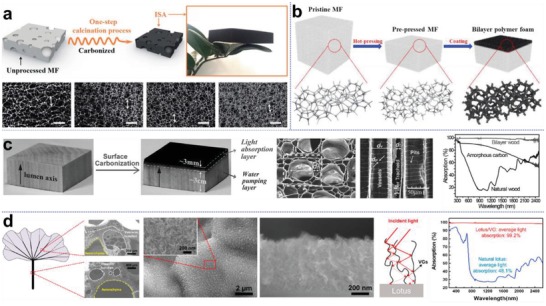
Commercial and natural hierarchical materials. a) Solar evaporator preparation by direct carbonization of melamine foam. SEM images shows shrunk pores were observed after calcination process, which would facilitate water transport and light absorption. Scale bar: 200 µm. Reproduced with permission.^[^ ^108^ ^]^ Copyright 2018, Royal Society of Chemistry. b) PPy modified melamine foam as bilayer solar absorber with broadband light absorption (94%). Reproduced with permission.^[^ ^88^ ^]^ Copyright 2019, Elsevier. c) Wood‐based solar absorber fabricated via simple surface carbonization. Due to the rich channeled mesostructures, carbonized wood is able to absorb nearly all the incident light over the entire wavelength range. Reproduced with permission.^[^ ^72^ ^]^ Copyright 2017, Wiley‐VCH. d) Hierarchical composite of vertically‐oriented graphene (VGs) on lotus substrate for light trapping and extremely high light absorption (99.2%). Reproduced with permission.^[^ ^113^ ^]^ Copyright 2019, Elsevier.

In addition to the synthetic materials, there are a wide range of commercially and naturally available hierarchical structures with superior light absorption that have been introduced as efficient solar evaporators. A typical example is melamine foam (MF), a porous material widely used in various research applications. By means of simple thermal treatment, MF can be readily converted into light‐absorbing carbon sponge (**Figure** **6**a), and the retrained porous structure not only facilitates light capturing (≈96%), but also enables rapid water transport.^[^ ^108^ ^]^ This versatile foam as substrate can also be integrated with other blackened materials like PPy as shown in Figure 6b to construct bilayer hierarchies for high light absorption.^[^ ^88^ ^]^ Hierarchal structures also widely exist in nature, and these natural materials that are now in widespread use as efficient solar evaporation devices, have become an integral branch in photothermal carbon‐based materials. Among them, wood materials, which are of particular interest, have been extensively investigated.^[^ ^39^, ^86^, ^109^, ^110^, ^111^, ^112^ ^]^ As shown in Figure 6c, a piece of wood block with hierarchical channeled structure after facile surface‐carbonization treatment is able to absorb 99% of light of wide spectrum.^[^ ^72^ ^]^ The excellent optical property of wood material along with its low thermal conductivity makes it an ideal photothermal material for efficient steam generation.^[^ ^72^, ^87^ ^]^ Aside from the direct carbonization treatment of natural materials, surface modification on the natural substrates for enhanced light absorption is also offers an effective approach. Recently, Ostrikov and co‐workers have demonstrated an interesting integrative structure by depositing vertically oriented graphenes (VGs) on a lotus micro‐textured surface (lotus/VG). The innate porous structure of lotus combined with the supplementary nanosheet coating facilitates better light capture, leading to a broadband light absorption, which is as high as 99.2%.^[^ ^113^ ^]^ Other essential factors that makes natural materials popularly considered as promising light absorbers include the great abundance and low cost. Hence, the natural solar evaporator materials hold enormous potential for future large‐scale applications.

### 2.4 Surface Heat Management

**Figure 7:**
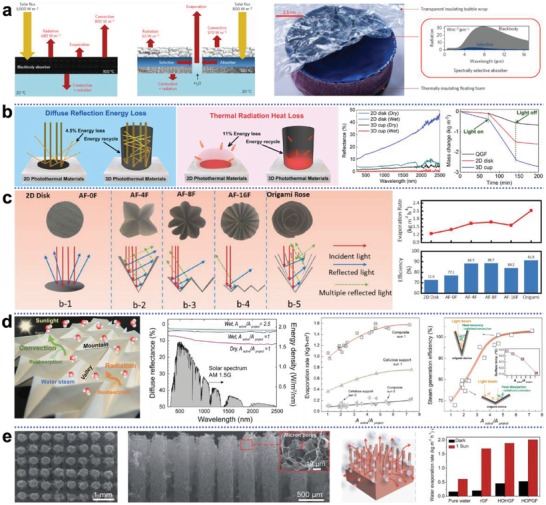
Surface heat management strategies for improved conversion efficiency. a) Replacing the blackbody absorber with a spectrally selective absorber, which has high solar absorptance and low thermal emittance is able to suppress radiative losses; a layer of bubble wrap covering on the absorber transmits sunlight, and minimizes convective losses. Reproduced with permission.^[^ ^20^ ^]^ Copyright 2016, Springer Nature. b) Surface heat confinement by using a 3D cup shaped solar absorber. The cup structure not only reduces light reflection, but also recovers radiation and convection heat losses through its wall. As a result, 99.4% of the incident light is absorbed and solar conversion efficiency close to 100% is achieved. Reproduced with permission.^[^ ^114^ ^]^ Copyright 2018, Elsevier. c) The origami inspired macrostructures (e.g., the origami rose) have larger surface area than 2D planar structure, and because of the significantly increased exposure area, the surface temperature tends to be lower than that of 2D disk structure, which would passively reduce heat loss to environment and thus increase conversion efficiency. Reproduced with permission.^[^ ^115^ ^]^ Copyright 2019, Wiley‐VCH. d) Origami structure at microscale. It is demonstrated that as the areal density (defined as the ratio of light interactive area to the projected area) increases, light absorption and evaporation performance of micro‐origami structure are both enhanced due to multiple reflection of light beam and radiation/convection heat recovery. Reproduced with permission.^[^ ^116^ ^]^ Copyright 2018, American Chemical Society. e) Vertically aligned graphene pillar array (HOPGF) allows pillars to mutually assimilate radiation/convention heat. Thermal dissipation to environment is therefore minimized, leading to higher evaporation rate. Reproduced with permission.^[^ ^117^ ^]^ Copyright 2018, Royal Society of Chemistry.

Heat loss to bulk water through downward conduction can be significantly reduced with thermal insulation, however, little is known in regard to approaches to reduce surface convection and radiation loss. To further improve conversion efficiency, surface heat losses must be suppressed. Chen and co‐workers revealed the severely adverse influence caused by surface heat loss with the energy balance and heat transfer diagram shown in **Figure** **7**a.^[^ ^20^ ^]^ To overcome it, they replaced the conventional blackbody absorber with a selective absorber with low thermal emittance, and the absorber was then covered by a sheet of transparent bubble wrap to minimize convective loss. With the reduced surface loss, this special design synergistically leads to a net improvement in steam generation. For blackbody solar absorber, strategies to reduce surface heat mainly focus on heat saving and recovery, which can be achieved by using macro‐ or micro‐3D photothermal structures.

A typical macro‐3D structure is the cup shaped photothermal material developed by Wang and his co‐workers.^[^ ^114^ ^]^ As shown in Figure 7b, compared to 2D planar structure in which surface heat freely dissipates, the 3D cup structure confines and recycles the convection and radiation heat within the cup walls, thus minimizing surface losses. The 3D structure exhibits enhanced light absorption, which leads to a conversion efficiency close to 100%. Origami provides very conducive inspirations in designing 3D materials for surface heat manipulations. The PPy‐based origami rose (Figure 7c), similar to the cone structures, is able to trap incident light, and it is interesting to note that, the increased actual exposure area reduces surface temperature, which would passively save heat loss to the environment.^[^ ^115^ ^]^ The origami approach is equally effective at microscale. It can be seen in Figure 7d that, by increasing the ratio of interactive area and projected area of micro‐origami device, the light absorption increases as a result of light trapping, while in the meantime, the periodically concave structure recovers radiative and convective heat, yielding an extraordinarily high conversion efficiency.^[^ ^116^ ^]^ Qu and co‐workers have developed a highly efficient structure of 95% efficiency that is composed of vertically aligned graphene pillar array (Figure 7e).^[^ ^117^ ^]^ Unlike previous evaporators in which the microstructures are water filled because of capillary effect, the novel array structure leaves free space between the graphene pillars. Convection and radiation heat between pillars can thereby be mutually assimilated and localized within the structure, instead of being lost to the environment.

The above cases indicate surface heat reduction is crucial for solar absorbers to realize efficiency close to the limit 100%, and it is concluded that, vertically organized structure at both macro and micro scale is believed to be highly conducive to surface heat management.

## 3 Enhanced Solar Evaporation

One implication of using solar energy for steam generation is that the evaporation process is not possible to transcend beyond its energy limit, which is capped by solar illumination, provided no optical concentration is involved. As the efficiency of well‐engineered photothermal materials developed so far approaches to 100%, there are pioneering attempts have been made to further enhance solar evaporation and achieve evaporation rate that could be even higher than the theoretical solar limit with unchanged projected area.

### 3.1 Enhancement from Environmental Heat

One approach that has been extensively explored is to introduce environmental heat into the solar evaporation system in a passive manner. To achieve this, two critical conditions have to be met: i) temperature difference between the system and its environment (which usually refers to surrounding air) should be created; ii) adequate interfacial area to take in environmental heat. Quite contrary to the traditional notion that radiated solar absorbers lose heat to the environment, heat can also be reversely transferred into the evaporation system via evaporative cooling effect, but it should be noted that it is the low‐temperature components (e.g., capillary wicks) instead of the hot evaporator that receives the extra heat input. Given that the capillary wick for water supply in a solar evaporation system are not exposed to the light, its temperature tends to be lower than that of its surroundings due to the aforementioned effect that heat is extracted from it via water evaporation from itself, thus leading to the built‐up of local temperature difference for environmental heat harvesting. The absorbed environmental heat is further used to drive more evaporation from these low‐temperature components in addition to the solar evaporators, as a result, the overall steam production is enhanced.

**Figure 8:**
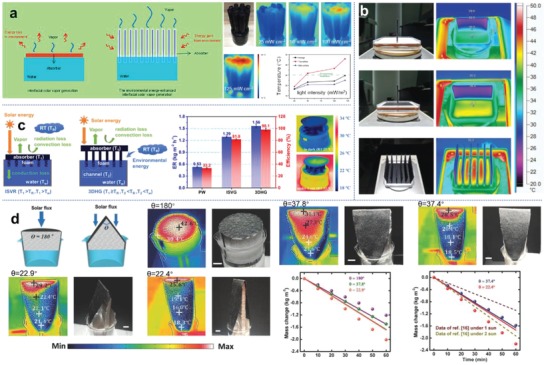
Photothermal structures to harvest environmental heat for enhancement of solar evaporation. a) Environmental energy absorbed from side surface of an interfacial solar vapor generator. The infrared images and graph show that under light illumination with various intensity, the overall temperature of solar evaporator is lower than the environmental temperature, allowing a reverse heat transfer from environment to solar evaporator. Reproduced with permission.^[^ ^118^ ^]^ Copyright 2018, Elsevier. b) Additional low‐temperature vertical evaporator array inserted on a solar absorber to confine surface heat and absorb energy from air. Reproduced with permission.^[^ ^119^ ^]^ Copyright 2018, Wiley‐VCH. c) Environmental heat harvesting by spatial water supply. It is shown that creating more exposed area during water transport not only leads to an increased actual area for evaporation, but also maintains the system's average temperature lower than room temperature, which would facilitate energy conduction from the ambience to evaporator. Reproduced with permission.^[^ ^120^ ^]^ Copyright 2019, American Chemical Society. d) A triangle photothermal structure for energy harvesting from warmer surroundings to generate cold vapor. By adjusting the apex angle, surface temperature of solar evaporator lower than that of its surroundings can be obtained. Reproduced with permission.^[^ ^121^ ^]^ Copyright 2018, Wiley‐VCH.

In a representative work conducted by Zhu and co‐workers,^[^ ^118^ ^]^ a bundle of cotton cores were employed to simultaneously harvest solar energy and environmental heat (**Figure** **8**a). The top surface localizes solar heat while heat from surrounding air is absorbed laterally. In another design as shown in Figure 8b, a solar absorbing plate is located at bottom and extra evaporative boards are vertically inserted into it for environmental heat harvesting.^[^ ^119^ ^]^ In both these designs, the wet components without light illumination are cooler than the environment due to the evaporative cooling effect, which allows for solar evaporation enhancement with the additional energy input from the warm air. Environmental heat can also be supplemented by solar absorbing surface through reverse convection and radiation. As shown in Figure 8c, a planar solar absorber is coupled with water transport channels, and the space gap between them makes the whole device directly exposed to warm air.^[^ ^120^ ^]^ The resulting average temperature of the 3D structure, including the solar absorber and water channels, is lower than the ambience, leading to reverse heat transfer from surroundings to the solar evaporation system. Song et al. proposed a straightforward method to achieve low‐temperature absorber by increasing the active surface area within a given projected area, with a triangle photothermal structure as illustrate in Figure 8d. It was observed that when the apex angle was reduced from planar 180° to 22.9°, temperature of major areas of the triangle structure dropped below the surrounding temperature, but evaporation rate increased up to 2.02 kg m^−2^ h^−1^, significantly higher than the solar limit of normal 2D planar absorber (1.68 kg m^−2^ h^−1^).^[^ ^121^ ^]^

A major concern that arises in this regard to this phenomenon is that in the previously published reports, (including the above highlighted examples) the solar evaporation device was considered as an open system wherein the capillary wicks and other components can interact with the ambient atmosphere, resulting in heat and mass transfer. However, in real applications, all the components of solar evaporation are enclosed within the solar still devices. The closed chamber would thus become a greenhouse or a closed system under light radiation. This raises questions whether the proposed mechanism of reverse heat transfer from the ambient to the solar evaporator can still work if it is a closed system.

### 3.2 Reduced Vaporization Enthalpy

**Figure 9:**
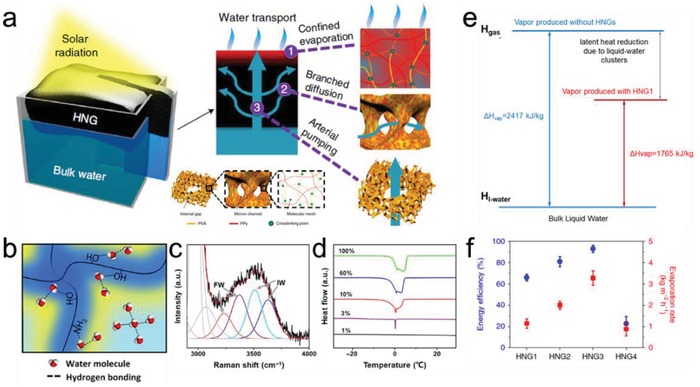
Reducing vaporization enthalpy to enhance steam generation. a) A hydrogel based photothermal evaporator with hierarchically porous structures (HNG). b) Different Water states in a hydratable polymer network, and the corresponding characterization of c) Raman spectra and d) DSC curves. e) Diagram showing the reduction in vaporization enthalpy. f) Solar evaporation performance of HNG. (a,e,f) Reproduced with permission.^[^ ^123^ ^]^ Copyright 2018, Springer Nature. (b–d) Reproduced with permission.^[^ ^124^ ^]^ Copyright 2019, American Association for the Advancement of Science.

Unlike traditional evaporation process where water changes its phase from liquid to vapor as individual molecules, Yu's group has recently disclosed a surprising and non‐intuitive water evaporation phenomenon by using a special water‐polymer network.^[^ ^62^, ^122^ ^]^ As shown in **Figure** **9**a, the developed polyvinyl alcohol (PVA) based hydrogel composite with a hierarchical nanostructure (HNG) confined water molecular meshes.^[^ ^123^ ^]^ The unique water–polymer interactions induced by the functional groups in polymer chains allows for the coexistence of water in different states, which includes bound water, intermediate water and free water (Figure 9b).^[^ ^124^ ^]^ In comparison to pure water in which the share of free water is dominant, more intermediate water is identified in the hydratable polymer networks via Raman spectroscopy and differential scanning calorimetry (DSC) measurements (Figure 9c,d). The increased fraction of intermediate water is crucial for enhancing water evaporation as it requires less energy than free water to realize phase‐change (Figure 9e). With the reduced vaporization enthalpy, the hydrogel‐based evaporator is able to produce significantly more water vapor under same solar input compared to other conventional photothermal materials (Figure 9f).

Although ultrahigh evaporation rate has been experimentally achieved in this water‐polymer network, the phenomenon is far from being fundamentally understood. By now the analysis on the distribution of intermediate water has given a reasonable explanation, however, its relationship with water cluster theory requires future definition. One possibility is both effects exist as it is observed that the salt ion concentrations in the condensed water were relatively high,^[^ ^125^ ^]^ which might be caused by the ion encapsulation in water clusters during evaporation. It is thereby of importance to ascertain the state of salty ions in the networks. Besides, how the different states of water influences the flow (diffusion and advection) in the hydrophilic hydrogel matrix is also a question requiring theoretical considerations.

Applications of this reduced vaporization enthalpy of water should not be redistricted to solar water production only. If the above findings could be finally applied in the real world, it would be revolutionary since our global energy production, which mostly rely on the conversion of heat into mechanical work by the phase‐change of water (Rankine cycle), will have a significant increase in efficiency.

### 3.3 Other Strategies

In addition to above approaches, there are also extraordinary strategies in development with impressively high evaporation rate demonstrated. The representative cases are highlighted in this section.

**Figure 10:**
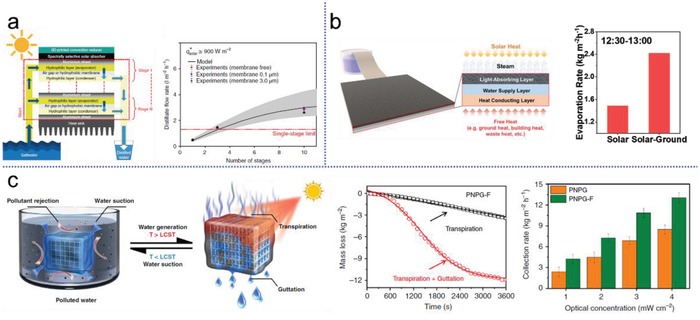
Strategies to achieve extra high solar evaporation rate. a) Latent heat recovery for multi‐stage steam generation using a passive solar distiller. A distillate flow rate of almost 3 L m^−2^ h^−1^ from seawater was demonstrated in a 10‐stage design. Reproduced with permission.^[^ ^84^ ^]^ Copyright 2018, Springer Nature. b) Solar evaporation enhanced by additional free energy. Trilayer photothermal structure (TLS) for hybrid energy harvesting. Reproduced with permission.^[^ ^126^ ^]^ Copyright 2019, American Chemical Society. c) Photothermal transpiration/guttation enhanced water production. The sunlight‐powered purifier (PNPG‐F) swells with water suction when its temperature is lower than the lower critical solution temperature (LCST). Direct light illumination increases its temperature with steam generation (transpiration), and when above LCST, water will be released from it via the guttation effect. Compared to conventional solar evaporation, the combination of transpiration and guttation endows the composite with a considerably higher water production rate (4.2 kg m^−2^ h^−1^). Reproduced with permission.^[^ ^127^ ^]^ Copyright 2019, Springer Nature.

As aforementioned, the generated hot water vapor contains a large amount of latent heat, which can be possibly reused multiple times for more steam generation before completely dissipated to the environment. Based on this idea, Asinari and co‐workers developed a passive multi‐stage solar distiller, as illustrated in **Figure** **10**a, for low‐cost and high‐yield seawater desalination.^[^ ^84^ ^]^ Water vapor produced by the top stage, which is heated by solar absorber, diffuses downward and condenses on the top of next stage, and by reusing the heat released from this ensuing condensation, secondary steam is generated. After 10 cycles of evaporation/condensation, the final water yield can reach up to 3 kg m^−2^ h^−1^, more than twice than that of conventional single stage evaporation. Different from the enhancement by passively harvesting environmental heat, hybrid heating initiatively makes use of all kinds of free heat including solar energy, surficial heat, waste heat etc., to achieve extra high evaporation rate. Tan and co‐workers recently proposed a trilayer photothermal structure for this purpose.^[^ ^126^ ^]^ Notably, a metal‐based heat conducting layer was employed as substrate to harvest free waste heat such as ground heat and waste heat from building wall, and the top surface can at the same time efficiently absorb light and release water vapor (Figure 10b). The practically obtained peak evaporation rate driven by surficial and solar heat was 2.4 kg m^−2^ h^−1^, almost double the yield under 1 sun illumination only. Besides, sunlight can simultaneously be used during solar evaporation to trigger beneficial effects (mechanical deformation, temperature response, wettability changes, etc.) with multifunctional materials to serve performance enhancement. Geng et al. recently demonstrated an interesting plant leaf inspired solar water purifier (Figure 10c), by means of transpiration/guttation effect triggered by sunlight, liquid clean water is repelled from the tailored PNPG‐F structure, offering an extremely high water collection of 4.2 kg m^−2^ h^−1^ under 1 sun.^[^ ^127^ ^]^

## 4 Salt Rejection Strategies for Long‐Term Solar Desalination

**Figure 11:**
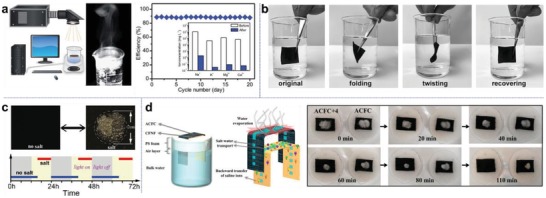
Salt removal by physical washing and natural dissolution. a) Laboratory experimental setup for continuous solar interfacial evaporation test. The graph demonstrates favorable stability of solar evaporator for sustainable desalination with no salt interference. Reproduced with permission.^[^ ^128^ ^]^ Copyright 2018, Royal Society of Chemistry. b) Demonstration of direct rinsing and washing of a highly flexible and robust membrane for salt removal. Reproduced with permission.^[^ ^111^ ^]^ Copyright 2017, Wiley‐VCH. c) Automatic dissolution of salt accumulated on a wood‐based solar absorber surface under dark conditions. Reproduced with permission.^[^ ^39^ ^]^ Copyright 2018, Wiley‐VCH. d) Enhanced salt dissolution by using an auxiliary backward water path. Photographs show that with extra water path (ACFC+4) salt dissolution is strengthened and takes less time. Reproduced with permission.^[^ ^92^ ^]^ Copyright 2019, American Chemical Society.

Solar interfacial evaporation is a powerful technology suitable for all types of undrinkable water purifications, and seawater desalination, universally known as one of the most promising solutions to address global water crisis, is mostly selected for purification demonstrations. However, desalination brings about a vital issue that interfacial evaporators have to deal with, which is salt accumulation and consequent deterioration in evaporative performance.^[^ ^25^ ^]^ Long‐term sustainable and high‐efficiency evaporation as the example shown in **Figure** **11**a, in which no significant efficiency decrease is observed over continuous operation, is always desired for ideal solar desalination.^[^ ^128^ ^]^ In this section, strategies for salt‐rejection in solar interfacial desalination process have been systematically summarized. Our findings indicate that the salt issue can be properly resolved with ingenious structures, and design principles to achieve it have been discussed.

### 4.1 Physical Removal and Natural Dissolution

When salt crystals form and accumulate on solar absorbers' surface, the primary method to remove it is physical cleaning, which refers to simple rinsing and washing.^[^ ^88^, ^93^, ^129^, ^130^, ^131^, ^132^, ^133^ ^]^ This method is particularly suited for flexible membrane‐type devices as demonstrated in Figure 11b,^[^ ^111^ ^]^ which are easy to clean and do not involve heavy intervention work compared to bulk materials.

Porous materials are more inclined to suffer from this issue due to impeded water transport, and if without this intervention, salt would easily pile up on evaporator surface. The most commonly used method to clear off the salt‐clogging surface is to turn off light and let the salt automatically dissolve back to bulk water reservoir (Figure 11c).^[^ ^39^ ^]^ From the realistic angle, natural dissolution is a feasible solution. The light on/off cycles can be perfectly fulfilled by the natural alternation of day and night. However, the gradual formation of salt on evaporator surface inevitably limits its active operation time during daytime. Liu and co‐workers recently reported a backward water transfer path (Figure 11d to dredge the salt produced by its upper evaporative fiber cloth (ACFC), and the bilayer structure can durably desalinate seawater with no salt fouling.^[^ ^92^ ^]^ This instructive design can be imitated for those salt‐fouling photothermal materials to accelerate salt dissolution during the dark time. However, the above two strategies are at root passive ways to reject salt, and operation duration of solar evaporators could be constrained in the daytime. By contrast, solar evaporators that can repel salt simultaneously along with highly efficient vapor production can save manpower and physical resources, and is believed to be of more practical value.

### 4.2 Enhanced Fluid Convection

Self‐regeneration, a property that enables solar evaporators to continuously function with no salt formation on the heating surface, becomes a favorable factor worth considering at the stage of material design and fabrication. One widely applicable method to obtain such ability is through enhanced fluid convection between solar evaporator and water reservoir, which can dilute the high concentration brine within the evaporating area before the precipitation of salt crystals takes place, and can be readily achieved with special photothermal structure design.

**Figure 12:**
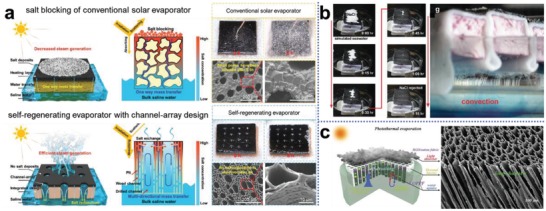
Enhanced fluid convection to facilitate salt exchange for self‐regenerating solar evaporators. a) Comparisons between conventional solar evaporator (poor fluid convection) and self‐regenerating evaporator (enhanced convection with millimeter‐sized drilled channels). Reproduced with permission.^[^ ^134^ ^]^ Copyright 2019, Wiley‐VCH. b) Demonstrations of a low‐cost floating solar evaporator that is able to prevent salt accumulation via enhanced water convection using fabric wick while simultaneously maintaining heat localization with thermal insulation. Reproduced with permission.^[^ ^136^ ^]^ Copyright 2018, Royal Society of Chemistry. c) Schematic and SEM images of vertically oriented porous polyacrylonitrile demonstrating strong water convection and salt exchange ability to prevent salt precipitation on upper evaporator. Reproduced with permission.^[^ ^137^ ^]^ Copyright 2019, Royal Society of Chemistry.

Hu's group reported a novel strategy to endow the solar evaporator with self‐regenerating ability by simply drilling millimeter‐sized channels within the naturally micro‐channeled structure of wood (**Figure** **12**a).^[^ ^134^, ^135^ ^]^ The millimeter‐scale drilled channels together with the microchannels of wood provide smoother pathways for rapid water transfer and solute exchange, thus leading to nil salt crystallization during intensive evaporation; by contrast, conventional solar evaporator that possess intricate internal structure can easily result in salt deposition on interface due to tortuous water transfer and confining convection. The rapid water flow not only accelerates salt exchange to avoid the formation of hypersaline water, but also takes away heat from the evaporation surface, which would cause extra conductive loss. At this point, Chen and co‐workers presented a salt‐rejecting fabric based solar evaporator that combined a wick structure for water supply and polystyrene foam as floating thermal insulation. This design as demonstrated in Figure 12b can spontaneously reject salt via enhanced fluid convection within the wick, and simultaneously maintain excellent heat localization.^[^ ^136^ ^]^ As such, the component responsible for water transport in a solar evaporation system greatly determines the salt‐rejection capability. Substrate with low‐tortuosity that is responsible for water supply such as the vertically oriented porous polyacrylonitrile foam (Figure 12c) is believed to be beneficial to fluid convection enhancement for salt rejection.^[^ ^137^ ^]^ At present, our understanding of water transport in these microstructures is still not sufficient. For example, advection and diffusion induced by salinity gradient can both contribute to salt rejection, but the underlying mechanism is still not clear. Numerical simulation could be a useful tool to help us gain a better understanding of water transport behavior in order to pursue a more efficient design of salt‐rejecting structures.^[^ ^136^ ^]^

### 4.3 Hydrophobic Design

**Figure 13:**
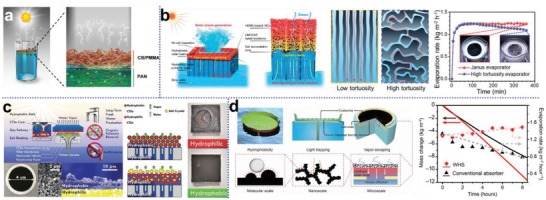
Hydrophobic design for salt rejection. a) Schematic of the salt resistant hydrophobic/hydrophilic bilayer Janus absorber. Reproduced with permission.^[^ ^141^ ^]^ Copyright 2018, Wiley‐VCH. b) Illustrations of low‐tortuosity and thermally insulative structure to improve stability and conversion efficiency of hydrophobic evaporator. Water supply and convection in a high‐tortuosity evaporator are impeded and thus the evaporator is more likely to result in deteriorated evaporation behavior. Reproduced with permission.^[^ ^142^ ^]^ Copyright 2019, Royal Society of Chemistry. c) Demonstrations of a thermal insulation coupled hydrophobic/hydrophilic solar evaporator. Due to the suppressed heat loss to bulk water by using thermal insulation and confined water supply, salt free and high‐efficiency (86.5%) evaporation can be achieved. Reproduced with permission.^[^ ^143^ ^]^ Copyright 2018, Royal Society of Chemistry. d) A water‐lily inspired salt‐rejecting structure. In this design, a thin water film is confined in the gap between the hydrophobic solar absorber and bulk water. The water film minimizes heat loss while ensuring sufficient water supply for stable evaporation. Reproduced with permission.^[^ ^144^ ^]^ Copyright 2019, American Association for the Advancement of Science.

The overwhelming majority of currently available solar evaporators are made of hydrophilic materials because of their advantage in water transport and hydrophobic materials are rarely used. As our understanding of photothermal structure deepens, the unique strengths of hydrophobic evaporators have been gradually recognized. The self‐healing hydrophobic structure that is usually coupled with a hydrophilic substrate for water pumping has been proved able to effectively avoid salt formation.^[^ ^138^, ^139^, ^140^ ^]^ The mechanism of salt‐rejection by hydroscopic/hydrophilic design is intelligible. As illustrated in **Figure** **13**a, the hydrophilic substrate (PAN layer) during steam generation is immersed under water, while the light‐absorbing hydroscopic layer (CB/PMMA), which is above the water, localizes solar heat and release water vapor. The salt ions are thereby blocked at the hydrophobic/hydrophilic interface and remains below the water level to eliminate any possibility of salt formation on CB/PMMA layer.^[^ ^141^ ^]^ However, the hydrophobic design has its own limits in the form of relatively low efficiency. Water phase change occurs at the interface of hydrophilic base by absorbing the heat from hot hydrophobic layer, but the direct immersion in bulk water dissipates the heat and leads to downward heat loss and reduction in photothermal conversion.

In response to this problem, it is necessary to introduce thermal insulation in the system to suppress conduction loss. The application of thermal insulation will more or less change the way of water supply, and it should be noted that salt precipitation might still occur if water fails to be delivered with low resistance. Hu et al. demonstrated a Janus evaporator with conversion efficiency of 80% achieved by combining hydrophilic backbone and hydroscopic coating.^[^ ^142^ ^]^ The backbone structure ensures rapid water supply for evaporation, and also thermally insulates heat from downward conduction. Effect of hydrophilic structure with different tortuosity of the water pumping structure on salt rejection was further studied. Their findings reveal that high‐tortuosity hydrophilic layer tends to result in salt formation, by comparison, low tortuosity can durably sustain evaporation process (Figure 13b). The mechanism, as we see, is similar to that as we previously discussed in fluid convection section, and equally applies to the hydroscopic strategy. In summary, hydrophilic substrate with straightforward internal structure to support hydrophobic solar absorber is of greater likelihood to avoid salt crystallization issue during its long‐term operation. Que and co‐workers developed a novel salt‐free hierarchical hydrophobic/hydrophilic bilayer evaporator.^[^ ^143^ ^]^ Solar conversion efficiency of 86.5% was achieved through complete isolation of the solar absorber from bulk water with additional thermal insulation layer and confined water supply (Figure 13c). Recently, Zhu and co‐workers presented a water lily‐inspired floating hierarchical structure (WHS) with impressive salt‐rejection capacity.^[^ ^144^ ^]^ In this design, a separate water layer between hydrophobic absorber and bulk water is created as illustrated in Figure 13d. With it, conduction heat loss is confined within the thin water layer, barring further loss to bulk water. Compared to conventional absorber whose evaporation rate degrades over time due to salt clogging phenomenon, WHS can stably maintain 80% efficiency and the its salt‐resistant property which enables itself to completely separate water and solute.

According to the examples as highlighted above, we have noted that almost all the hydroscopic solar absorbers have a planar configuration, but macro‐3D designs like cone‐shape, origami‐like structures that are able to suppress surface heat loss and increase overall evaporation rate have not been explored. Such a limitation is a result of the restricted water supply in hydrophobic evaporators that would force solar absorbers to function in close proximity to water source, but there is still a substantial possibility to achieve it, as long as the hydrophilic substrate soundly attaches to the hydrophobic layer and transfers water with strong convection.

### 4.4 Site‐Specific Salt Formation

Site‐specific salt formation for self‐regenerating solar evaporation is a recently emerging strategy, it can be defined as follows: water flux, mostly driven by capillary force, carries salt ions from water source to a specific site, e.g., the edges of solar absorbers. The solute concentration increases as the water flux nears the terminus, as a result of water loss across the evaporative area. Interestingly, salt crystallization takes place only at the terminal site because of the directional water flow, thus leaving the major area of solar evaporators free from salt issue. This manipulated salt distribution can simultaneously sustain water evaporation while effectively extracting salt from brine.

**Figure 14:**
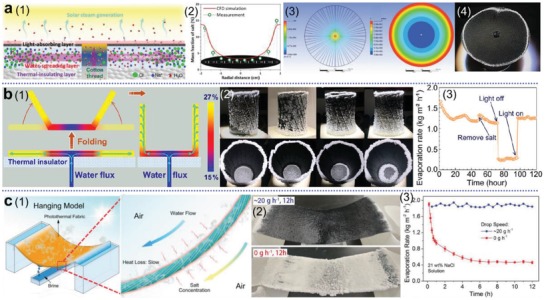
Site‐specific salt formation for salt‐rejection on evaporation area. a): 1) Illustration of water and ion transport in the solar steam generator. 2) Salinity distribution of the circular evaporation disc. Results show that salt ions are conveyed and finally crystalizes at the edge region. 3) Computational fluid dynamics (CFD) simulation and 4) photograph of experimental result of a one‐inlet configuration. Reproduced with permission.^[^ ^145^ ^]^ Copyright 2019, Royal Society of Chemistry. b): 1) Water transport route in a 3D cup configuration. 2) Photographs of salt formation on the 3D cup solar evaporator. 3) Water evaporation rate of the cup shaped evaporator under 1 sun illumination for long‐term brine treatment. Reproduced with permission.^[^ ^146^ ^]^ Copyright 2018, American Chemical Society. c): 1) Schematic illustration of a hanging evaporator and its water flow. 2) Controllable salt formation by adjusting drop speed. 3) Drop speed controlled stable evaporation. Evaporation rates of the hanging fabric evaporator at different drop speed. Reproduced with permission.^[^ ^147^ ^]^ Copyright 2019, Wiley‐VCH.

Recently, Zhang and co‐workers have presented a representative approach to direct salt formation and maintain evaporation performance, by adjusting the position of water feeding cotton threads to control the direction of water flow (**Figure** **14**a).^[^ ^145^ ^]^ As revealed from the results of salt concentration gradient and corresponding simulation using computational fluid dynamics (CFD), salt ions are carried by water flux from the center of a circular solar absorber radially to its edge, and salt forms and accumulates only within the edge area, leaving the main evaporation area evaporation unaffected. This strategy is also of good compatibility for 3D configuration as the example given in Figure 14b. Water is delivered to a 3D cup‐shape evaporator through a 1D water pathway, and because of the unidirectional water flow, salt is apt to precipitate at the cup rim and curvilinear wall, so that the light absorbing bottom area remains salt‐free and maintains its evaporation rate.^[^ ^146^ ^]^ However, regular removal is needed because the falling salt clusters might block the bottom area. In addition to capillary suction, the direction of water flow can also be regulated by other forces like gravity and using hydraulics. In a recent study carried out by Liu and co‐workers as shown in Figure 14c, a durable hanging photothermal structure was demonstrated. Driven by capillary and hydraulic force, saline water flows from two ends of a fabric evaporator to its low‐lying center and subsequently detaches from the device by dripping. By increasing the drop rate, which is controlled by hydraulic head difference, brine dripping are able to bring away salt and hence allow evaporation process to continue without inhibition caused by salt accumulation.^[^ ^147^ ^]^

Till now, there are only a few reports of solar evaporators for sustainable desalination following the site‐specific salt formation strategy. At the moment, imperfections of this method have been preliminarily identified. It can be safely inferred that for long‐term operation, threat of salt accumulation at the margin of solar absorbers to the evaporating area would grow if the solid salt is not removed frequently. In the case of water flow driven by hydraulic force, the force has to be maintained by adjusting the hydraulic head difference from time to time. Current understanding of this interesting strategy to repel salt is still not adequate, and future modifications can be possibly achieved to address the above concerns.

## 5 Perspectives

### 5.1 Benefits of Vertical Architectures

Our investigations reveal that the structure of photothermal materials affects almost every aspect of steam generation, and it plays a decisive role in deciding certain properties such as optical absorption and salt‐rejection ability. In particular, we wish to emphasize the importance of 3D vertical structures. Based on the highlighted examples in this review, we find that the vertical structures, either at microscale or macroscale, are believed to have the most beneficial impact on the evaporation performance.

At micrometer and nanometer scale, vertically hierarchical structures not only enhance light absorption through light‐trapping effect, in some cases, vertically aligned structures such low‐tortuosity channels can also facilitate fluid convection to prevent salt precipitation. Additionally, vertical array structures have also been demonstrated to suppress surface heat loss via mutual assimilation of convection and radiation heat. Macroscale structures such as origami inspired and cup‐shape evaporators have been proved capable of confining surface heat to reduce radiation and convection loss. Vertically increasing the scale of solar evaporators can possibly enable environmental heat harvesting to achieve ultrahigh‐rate evaporation beyond solar limit. As far as the efficiency is concerned, vertical design for solar evaporator architecting is preferable over planar configuration. However, from a practical perspective, 3D macro‐sized solar evaporators will undoubtedly increase the consumption of raw materials, transportation cost and installation complexity. The catch, however, is that by how much will the 3D architecture exceed the performance of a conventional 2D device if put into operation in a realistic operational condition and whether the extra cost spent on the development and construction of the 3D evaporator and its maintenance can be justified over time.

### 5.2 Fundamental Understanding of Solar Desalination System

One of the major impediments to the development of solar desalination is, not all the previous reports used seawater for water purification demonstration, and as a result, whether the reported evaporators are competent to continuously and stably desalinate saline water remains to be seen. But these studies are of great value to facilitate our understanding of efficient photothermal conversion. Another issue is we still lack a comprehensive interpretation of water transfer and strong evidence of water path in wicking materials. It is bewilderingly observed in some cases with confined water path that no salt precipitation occurs, but others reveal that the confined water supply gives rise to massive salt accumulation on the solar absorber material. Reasons accounting for these observations are contrary to each other. The former emphasizes the role of fluid convection, which means water flows bidirectionally in the system, however, the latter claims that the salt is caused by one‐way water flow, i.e., unidirectional water supply. Both are correct and have been corroborated by respective experimental results, and the opposite observations can thereby be attributed to the different net water flux in each of these cases, which might be mainly controlled by the size and structure of wicking materials. However, no studies have tried to find evidences except for direct observation to disclose how mass including water and ions are transported and exchanged. With a better understanding of it, salt‐resistant evaporation system can be prepensely designed. Moreover, although there is an increasing number of demonstrations of high‐efficiency solar evaporators, a uniform protocol for systematic evaluation of solar conversion efficiency is still lacking so far for indoor testing.^[^ ^148^ ^]^ For example, as we have discussed in the section of Energy Balance, a broad consensus of the selection of enthalpy for a solar evaporator to calculate efficiency is yet to be reached.^[^ ^56^, ^81^, ^82^, ^115^, ^149^ ^]^ It is therefore suggested that, future research exploration on the fundamental understanding of solar interfacial evaporation is imperative for our community to evaluate the parameters and efficiency more effectively and accurately.

### 5.3 In Situ Energy Generation

**Figure 15:**
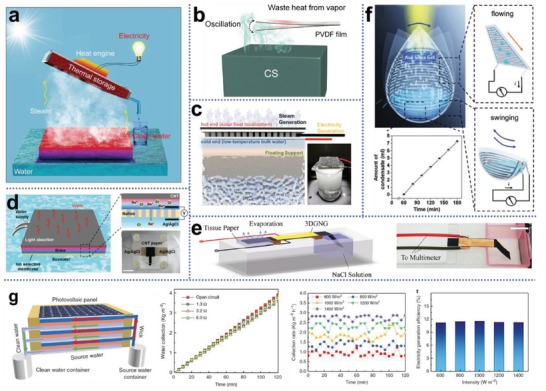
Energy generation from solar evaporation system. a) Storage and recycling of thermal energy of hot water vapor by a thermoelectric module. Reproduced with permission.^[^ ^83^ ^]^ Copyright 2018, Elsevier. b) Thermomechanical recovery through piezo‐pyroelectric by a fluoropolymer polyvinylidene fluoride (PVDF). Reproduced with permission.^[^ ^66^ ^]^ Copyright 2018, Wiley‐VCH. c) Thermoelectric power production making use of innate temperature difference between solar absorber and bulk water. Reproduced with permission.^[^ ^79^ ^]^ Copyright 2019, Elsevier. d) Salinity difference induced electricity generation. Reproduced with permission.^[^ ^153^ ^]^ Copyright 2017, Royal Society of Chemistry. e) Capillary‐driven power generation during water transport for evaporation. Reproduced with permission.^[^ ^155^ ^]^ Copyright 2019, American Chemical Society. f) Triboelectric generation during water condensation and droplet collection. Reproduced with permission.^[^ ^37^ ^]^ Copyright 2018, Wiley‐VCH. g) Multistage solar photovoltaic membrane distillation for Simultaneous production of fresh water and electricity. Reproduced with permission.^[^ ^159^ ^]^ Copyright 2019, Springer Nature.

Evaporation is a natural and ubiquitous phenomenon occurring ceaselessly, but the interfacial evaporation system is exceedingly complicated, involving varying temperature, phase change, mass transfer, etc. As previously discussed, research opportunities are intertwined with every link of evaporation process, and to identify them, the most effective way is to seek from the energy balance diagram of this system. Interesting attempts have been made recently. For example, the heat carried by hot water vapor can be recycled for electricity generation via thermoelectric (**Figure** **15**a) and thermomechanical effects (Figure 15b),^[^ ^66^, ^83^ ^]^ or directly utilized for rapid sterilization.^[^ ^81^, ^150^, ^151^, ^152^ ^]^ Converting downward heat loss into electricity has also been demonstrated through thermoelectric method as shown in Figure 15c, which is achieved by taking the advantage of inherently established temperature difference between hot solar absorber and cool water body.^[^ ^68^, ^79^, ^85^ ^]^

If we assume that water vapor is the staple product of solar desalination systems, the other underlying by‐products can also be made use of for energy‐related applications. It is observed that as pure water is extracted from seawater by rapid evaporation, a salinity gradient is created naturally between surface water and bulk seawater, which allows electricity generation by the concentration difference (Figure 15d).^[^ ^153^ ^]^ Studies have demonstrated that water flow for evaporation can induce electric current, which is illustrated in Figure 15e, and produce hydrovoltaic energy.^[^ ^154^, ^155^ ^]^ In addition to this, energy it is also possible to generate energy in the condensation of water vapor by using a triboelectric generator (Figure 15f).^[^ ^37^ ^]^ Another recent report has revealed that photothermal effect has the ability to enhance capacitance of supercapacitors.^[^ ^156^ ^]^ By combining with hydroscopic materials such as the hydrogel demonstrated by Tan and co‐workers recently, the generated water vapor was split into hydrogen and oxygen in an hybrid artificial photocatalysis system.^[^ ^157^, ^158^ ^]^ In this view, although there seems to be not much margin for improvement of conversion efficiency of solar evaporation, it has exhibited promising potential in playing a role to facilitate interesting and constructive interdisciplinary studies between different energy application fields. In addition to the interesting interdisciplinary combinations with solar evaporation, practical feasibility of these integrated systems should also command our attention. It hard to deny that a commercial solar panel can easily outperform the highlighted examples in terms of both power output and stability. For example, the cases highlighted above can hardly yield output power density of 1 W m^−2^ under 1 sun irradiation, in which the solar energy conversion efficiency would be less than 1%, whereas, commercially available solar panels could be 3 orders of magnitude higher, and particularly, 6 orders of magnitude larger than the piezo‐pyroelectric generator as shown in Figure 15b for waste energy recovery from generated water vapor. This direct comparison suggests that at this stage, these demonstrated electricity generators based on solar evaporation process can hardly make a difference in meeting the current or future energy trends. The limited power produced simultaneously with steam generation seems more likely to be useful to drive small electronics and devices, and it is thereby suggested that future energy generators integrated in solar evaporation systems should not only possess good scalability, but also focus more on practical applications. If we combine solar evaporation and electricity generation in a reverse way, which is to produce clean water in the process of traditional photovoltaic (PV) generation, more substantial gains would be possible. In a recent report, Wang and co‐workers have demonstrated such an integration, in which multi‐stage membrane distillation (MSMD) device was mounted at the backside of a solar cell to recycle wasted heat from solar panels for water distillation.^[^ ^159^ ^]^ Notably, PV performance suffered no compromise while water production rate of >1.64 kg m^−2^ h^−1^ was achieved (Figure 15g), which is already higher than most solar evaporators reported to date. This strategy realizes simultaneous electricity and water production, and more importantly, the gap between lab‐scale study and practical applications can be hopefully overcome in a few short years.

### 5.4 Practical Implementation

**Figure 16:**
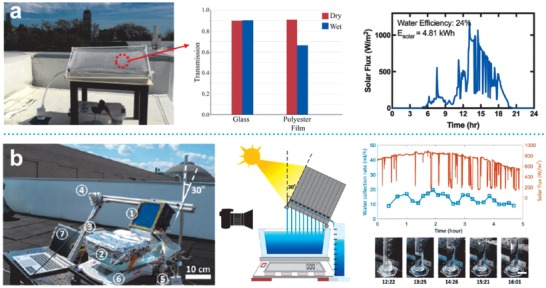
Outdoor tests of solar still systems for water desalination. a) Rooftop experiment of a floating solar still with polyester film cover. Reproduced with permission.^[^ ^136^ ^]^ Copyright 2018, Royal Society of Chemistry. b) Rooftop experiment of a multistage solar still with latent heat recovery. Reproduced with permission.^[^ ^160^ ^]^ Copyright 2018, Royal Society of Chemistry.

The underlying differences between practical implementation of solar evaporation systems and indoor laboratory measurements should be carefully considered prior to implementation of these systems for commercial applications in the near future. Particularly for long‐term desalination process, self‐regenerating stability should be considered as a criterion of equal importance to efficiency to evaluate a solar evaporation system in actual operation. Also, the condensation process is overlooked. Indoor testing is mostly carried out in an open configuration, by contrast, solar distillation device is a closed chamber and has to be coupled with a cover to effectuate water condensation and collection, and in this case, water droplet formation on the cover inevitably results in optical loss. As the rooftop test shown in **Figure** **16**a, the transparent cover (e.g., polyester film) of a solar still device lost over 20% transmission due to water condensation on it.^[^ ^136^ ^]^ Besides, vapor pressure and obliterative airflow in a closed evaporation‐condensation chamber that are likely to bring down evaporation rate should also be carefully considered from a realistic point of view.^[^ ^83^, ^115^ ^]^ However, the above issues have not yet been considered or systematically evaluated in most previous studies. As a result, the on‐site conversion efficiency and daily water production (≈2.5 kg m^−2^ day^−1^) of solar still would be far lower than the observations under ideal laboratory conditions (Figure 16a).

In a recent report led by Lin and co‐workers, more insights on the specific water productivity of solar thermal systems was provided.^[^ ^7^ ^]^ Their analysis has disclosed that PV–driven reverse osmosis can yield substantially more water per solar radiation area compared to the current solar‐driven desalination, and it also suggests that water productivity of solar desalination system could be effectively enhanced by maximizing latent heat recovery. As we already mentioned above, this approach has indeed exhibited great competence to enhance steam generation. With it, Wang and co‐workers in a latest demonstration have achieved an extremely high evaporation rate (2.6 kg m^−2^ h^−1^) by using a multistage solar still in a rooftop test (Figure 16b), which is almost comparable with one day's production of the conventional solar still as highlighted above.^[^ ^160^ ^]^ Although solar evaporator has the merits of broadband light absorption and capability of high‐salinity water desalination, the relatively low water productivity would make it much less competitive in the future desalination market. In this respect, latent heat recovery should be seriously taken into considerations when designing photothermal structures.

On material level, cost has been of primary concern. Cost effective manufacturing of solar absorbers is a prerequisite for a sustainable large‐scale application. Other costs induced during transportation, installation and operation should not be overlooked. Lightweight solar absorbers with good flexibility such as 2D membrane type solar absorbers are believed to be beneficial to reduce these costs by simplifying installation and maintenance process when compared to bulk and heavy materials. Portable and foldable solar evaporators can also befit small‐scale mobile water purification for personal and domestic use.^[^ ^126^ ^]^ The harsh marine environment might also pose an enormous threat against long‐term stability. Long periods of direct soaking in seawater may lead to the growth of micro‐organisms like algae within the solar absorbers, which would obstruct the path for water supply. Biofilm formation on evaporating surface could dramatically reduce light absorption and degrade evaporation behavior. To mitigate these, research attention on anti‐biofouling structures should be paid.^[^ ^161^, ^162^ ^]^ Decay is very likely to occur to photothermal materials with low biological resistance. Wood, which has been widely considered as one of the most promising solutions for large‐scale low‐cost solar desalination, is such a material suffering a high risk of decay in seawater over time. This is mainly attributed to the partial carbonizing process that is done on the surface of the wood to increase its light absorbing capability. Whilst only the surface of the wood is carbonized, the bulk wood body remains natural and untreated, making the wood‐based solar evaporators vulnerable to biological erosion. A recent report led by Tan and co‐workers has demonstrated that fully carbonized wood exhibits better biostability and higher evaporation rate than surface carbonized wood.^[^ ^25^ ^]^ To conclude, practical implementation of solar evaporators involves obstacles that can hardly be foreseen in laboratory, and therefore needs more pragmatic exploration in the future.

## 6 Conclusion and Outlook

Due to the dedicated efforts of researchers and experts from many different disciplines and fields, tremendous advances on the topic of solar interfacial evaporation have been made in past few years. The clearest example is the big leap in solar‐to‐steam conversion efficiency. Currently, there is an increasing number of photothermal materials and structures that have been demonstrated with an efficiency higher than 90%, which was scarcely possible earlier. Such an accomplishment is mainly attributed to the progress obtained in new photothermal structures, effective heat management and water supply strategies. Behind these demonstrations of high‐efficiency solar evaporation systems, systematic investigations of how the structure of a photothermal material affects its evaporation behavior and final conversion efficiency are still lacking. In this review, we have made such an attempt to look in detail at the role and function of material structures in efficient photothermal conversion process.

However, essential understanding of interfacial evaporation system is deficient. For example, the dissonance in measuring and reporting the solar conversion efficiency is one noticeable backlog in this area, which needs our immediate attention. Furthermore, discrepancies in the results obtained at lab scale and realistic conditions warrant that practical studies involving on‐field conditions is necessary before the concept of solar steam generation is proposed as a viable and sustainable method for desalination in the near future.

## Conflict of Interest

The authors declare no conflict of interest.

## References

[1] M. M. Mekonnen , A. Y. Hoekstra , Sci. Adv. 2016, 2, 1500323. 10.1126/sciadv.1500323 PMC4758739 26933676

[2] N. Ghaffour , T. M. Missimer , G. L. Amy , Desalination 2013, 309, 197.

[3] I. C. Karagiannis , P. G. Soldatos , Desalination 2008, 223, 448.

[4] P. Tao , G. Ni , C. Song , W. Shang , J. Wu , J. Zhu , G. Chen , T. Deng , Nat. Energy 2018, 3, 1031.

[5] S. Cao , Q. Jiang , X. Wu , D. Ghim , H. Gholami Derami , P.‐I. Chou , Y.‐S. Jun , S. Singamaneni , J. Mater. Chem. A 2019, 7, 24092.

[6] D. K. Nandakumar , Y. Zhang , S. K. Ravi , N. Guo , C. Zhang , S. C. Tan , Adv. Mater. 2019, 31, 1806730. 10.1002/adma.201806730 30637806

[7] Z. Wang , T. Horseman , A. P. Straub , N. Y. Yip , D. Li , M. Elimelech , S. Lin , Sci. Adv. 2019, 5, eaax0763. 31360770 10.1126/sciadv.aax0763 PMC6660204

[8] H. Sharon , K. S. Reddy , Renewable Sustainable Energy Rev. 2015, 41, 1080.

[9] Y. Zhang , M. Sivakumar , S. Yang , K. Enever , M. Ramezanianpour , Desalination 2018, 428, 116.

[10] T. Arunkumar , K. Raj , D. Dsilva Winfred Rufuss , D. Denkenberger , G. Tingting , L. Xuan , R. Velraj , Renewable Sustainable Energy Rev. 2019, 101, 197.

[11] T. P. Otanicar , P. E. Phelan , R. S. Prasher , G. Rosengarten , R. A. Taylor , J. Renewable Sustainable Energy 2010, 2, 033102.

[12] O. Neumann , A. S. Urban , J. Day , S. Lal , P. Nordlander , N. J. Halas , ACS Nano 2013, 7, 42. 23157159 10.1021/nn304948h

[13] E. T. Ulset , P. Kosinski , Y. Zabednova , O. V. Zhdaneev , P. G. Struchalin , B. V. Balakin , Nano Energy 2018, 50, 339.

[14] G. Ni , N. Miljkovic , H. Ghasemi , X. Huang , S. V. Boriskina , C.‐T. Lin , J. Wang , Y. Xu , M. M. Rahman , T. Zhang , G. Chen , Nano Energy 2015, 17, 290.

[15] H. Ghasemi , G. Ni , A. M. Marconnet , J. Loomis , S. Yerci , N. Miljkovic , G. Chen , Nat. Commun. 2014, 5, 4449. 25043613 10.1038/ncomms5449

[16] Z. Wang , Y. Liu , P. Tao , Q. Shen , N. Yi , F. Zhang , Q. Liu , C. Song , D. Zhang , W. Shang , T. Deng , Small 2014, 10, 3234. 24821378 10.1002/smll.201401071

[17] C. Chen , Y. Kuang , L. Hu , Joule 2019, 3, 683.

[18] L. Zhou , Y. Tan , D. Ji , B. Zhu , P. Zhang , J. Xu , Q. Gan , Z. Yu , J. Zhu , Sci. Adv. 2016, 2, e1501227. 27152335 10.1126/sciadv.1501227 PMC4846456

[19] K. Bae , G. Kang , S. K. Cho , W. Park , K. Kim , W. J. Padilla , Nat. Commun. 2015, 6, 10103. 26657535 10.1038/ncomms10103 PMC4682046

[20] G. Ni , G. Li , S. V. Boriskina , H. Li , W. Yang , T. Zhang , G. Chen , Nat. Energy 2016, 1, 16126.

[21] X. Wu , G. Chen , W. Zhang , X. Liu , H. Xu , Adv. Sustainable Syst. 2017, 1, 1700046.

[22] Y. Wang , H. Liu , C. Chen , Y. Kuang , J. Song , H. Xie , C. Jia , S. Kronthal , X. Xu , S. He , L. Hu , Adv. Sustainable Syst. 2019, 3, 1800055.

[23] T. Li , H. Liu , X. Zhao , G. Chen , J. Dai , G. Pastel , C. Jia , C. Chen , E. Hitz , D. Siddhartha , R. Yang , L. Hu , Adv. Funct. Mater. 2018, 28, 1707134.

[24] J. Zeng , Q. Wang , Y. Shi , P. Liu , R. Chen , Adv. Energy Mater. 2019, 9, 1900552.

[25] Y. Zhang , S. K. Ravi , S. C. Tan , Adv. Sustainable Syst. 2019, 3, 1900044.

[26] Z. Li , C. Wang , J. Su , S. Ling , W. Wang , M. An , Sol. RRL 2019, 3, 1800206.

[27] J. Zhou , Y. Gu , P. Liu , P. Wang , L. Miao , J. Liu , A. Wei , X. Mu , J. Li , J. Zhu , Adv. Funct. Mater. 2019, 29, 1903255.

[28] M. Gao , L. Zhu , C. Peh , G. W. Ho , Energy Environ. Sci. 2019, 12, 841.

[29] B. Gong , H. Yang , S. Wu , G. Xiong , J. Yan , K. Cen , Z. Bo , K. Ostrikov , Nano‐Micro Lett. 2019, 11, 51. 10.1007/s40820-019-0281-1 PMC7770882 34137985

[30] M. S. Zielinski , J. W. Choi , T. La Grange , M. Modestino , S. M. H. Hashemi , Y. Pu , S. Birkhold , J. A. Hubbell , D. Psaltis , Nano Lett. 2016, 16, 2159. 26918518 10.1021/acs.nanolett.5b03901

[31] M. L. Brongersma , N. J. Halas , P. Nordlander , Nat. Nanotechnol. 2015, 10, 25. 25559968 10.1038/nnano.2014.311

[32] J. A. Webb , R. Bardhan , Nanoscale 2014, 6, 2502. 24445488 10.1039/c3nr05112a

[33] Z. W. Seh , S. Liu , M. Low , S.‐Y. Zhang , Z. Liu , A. Mlayah , M.‐Y. Han , Adv. Mater. 2012, 24, 2310. 22467121 10.1002/adma.201104241

[34] M. Kaur , S. Ishii , S. L. Shinde , T. Nagao , Adv. Sustainable Syst. 2019, 3, 1800112.

[35] L. Tian , J. Luan , K. Liu , Q. Jiang , S. Tadepalli , M. K. Gupta , R. R. Naik , S. Singamaneni , Nano Lett. 2016, 16, 609. 26630376 10.1021/acs.nanolett.5b04320

[36] F. S. Awad , H. D. Kiriarachchi , K. M. AbouZeid , U. Ozgur , M. S. El Shall , ACS Appl. Energy Mater. 2018, 1, 976.

[37] M. Gao , C. K. Peh , H. T. Phan , L. Zhu , G. W. Ho , Adv. Energy Mater. 2018, 8, 1800711.

[38] Z. Sun , J. Wang , Q. Wu , Z. Wang , Z. Wang , J. Sun , C.‐J. Liu , Adv. Funct. Mater. 2019, 29, 1901312.

[39] M. Zhu , Y. Li , F. Chen , X. Zhu , J. Dai , Y. Li , Z. Yang , X. Yan , J. Song , Y. Wang , E. Hitz , W. Luo , M. Lu , B. Yang , L. Hu , Adv. Energy Mater. 2018, 8, 1701028.

[40] X. Wang , Y. He , X. Liu , G. Cheng , J. Zhu , Appl. Energy 2017, 195, 414.

[41] G. Song , Y. Yuan , J. Liu , Q. Liu , W. Zhang , J. Fang , J. Gu , D. Ma , D. Zhang , Adv. Sustainable Syst. 2019, 3, 1900003.

[42] M. Kaur , S. Ishii , S. L. Shinde , T. Nagao , ACS Sustainable Chem. Eng. 2017, 5, 8523.

[43] L. Zhou , Y. Tan , J. Wang , W. Xu , Y. Yuan , W. Cai , S. Zhu , J. Zhu , Nat. Photonics 2016, 10, 393.

[44] N. M. Haegel , Appl. Phys. A: Solids Surf. 1991, 53, 1.

[45] J. Wang , Y. Li , L. Deng , N. Wei , Y. Weng , S. Dong , D. Qi , J. Qiu , X. Chen , T. Wu , Adv. Mater. 2017, 29, 1603730. 10.1002/adma.201603730 27862379

[46] H. Liu , C. Chen , H. Wen , R. Guo , N. A. Williams , B. Wang , F. Chen , L. Hu , J. Mater. Chem. A 2018, 6, 18839.

[47] X. Li , Z. Yao , J. Wang , D. Li , K. Yu , Z. Jiang , ACS Appl. Energy Mater. 2019, 2, 5154.

[48] G. Zhu , J. Xu , W. Zhao , F. Huang , ACS Appl. Mater. Interfaces 2016, 8, 31716. 27801572 10.1021/acsami.6b11466

[49] R. Chen , Z. Wu , T. Q. Zhang , T. Yu , M. Ye , RSC Adv. 2017, 7, 19849.

[50] Y. Geng , K. Zhang , K. Yang , P. Ying , L. Hu , J. Ding , J. Xue , W. Sun , K. Sun , M. Li , Carbon 2019, 155, 25.

[51] L. Zhu , M. Gao , C. K. N. Peh , G. W. Ho , Mater. Horiz. 2018, 5, 323.

[52] A. B. Kuzmenko , E. van Heumen , F. Carbone , D. van der Marel , Phys. Rev. Lett. 2008, 100, 117401. 18517825 10.1103/PhysRevLett.100.117401

[53] X. Li , W. Xu , M. Tang , L. Zhou , B. Zhu , S. Zhu , J. Zhu , Proc. Natl. Acad. Sci. USA 2016, 113, 13953. 27872280 10.1073/pnas.1613031113 PMC5150409

[54] X. Hu , W. Xu , L. Zhou , Y. Tan , Y. Wang , S. Zhu , J. Zhu , Adv. Mater. 2017, 29, 1604031. 10.1002/adma.201604031 27885728

[55] Y. Ito , Y. Tanabe , J. Han , T. Fujita , K. Tanigaki , M. Chen , Adv. Mater. 2015, 27, 4302. 26079440 10.1002/adma.201501832

[56] Q. Jiang , L. Tian , K. Liu , S. Tadepalli , R. Raliya , P. Biswas , R. R. Naik , S. Singamaneni , Adv. Mater. 2016, 28, 9400. 27432591 10.1002/adma.201601819

[57] P. Zhang , J. Li , L. Lv , Y. Zhao , L. Qu , ACS Nano 2017, 11, 5087. 28423271 10.1021/acsnano.7b01965

[58] H. Ren , M. Tang , B. Guan , K. Wang , J. Yang , F. Wang , M. Wang , J. Shan , Z. Chen , D. Wei , H. Peng , Z. Liu , Adv. Mater. 2017, 29, 1702590. 10.1002/adma.201702590 28833544

[59] J. Yang , Y. Pang , W. Huang , S. K. Shaw , J. Schiffbauer , M. A. Pillers , X. Mu , S. Luo , T. Zhang , Y. Huang , G. Li , S. Ptasinska , M. Lieberman , T. Luo , ACS Nano 2017, 11, 5510. 28511003 10.1021/acsnano.7b00367

[60] Y. Li , T. Gao , Z. Yang , C. Chen , W. Luo , J. Song , E. Hitz , C. Jia , Y. Zhou , B. Liu , B. Yang , L. Hu , Adv. Mater. 2017, 29, 1700981. 10.1002/adma.201700981 28470982

[61] K. Kim , S. Yu , C. An , S.‐W. Kim , J.‐H. Jang , ACS Appl. Mater. Interfaces 2018, 10, 15602. 29667401 10.1021/acsami.7b19584

[62] X. Zhou , F. Zhao , Y. Guo , Y. Zhang , G. Yu , Energy Environ. Sci. 2018, 11, 1985.

[63] Q. Hou , C. Xue , N. Li , H. Wang , Q. Chang , H. Liu , J. Yang , S. Hu , Carbon 2019, 149, 556.

[64] P. Mu , Z. Zhang , W. Bai , J. He , H. Sun , Z. Zhu , W. Liang , A. Li , Adv. Energy Mater. 2019, 9, 1802158.

[65] G. Liu , J. Xu , K. Wang , Nano Energy 2017, 41, 269.

[66] L. Zhu , M. Gao , C. K. N. Peh , X. Wang , G. W. Ho , Adv. Energy Mater. 2018, 8, 1702149.

[67] Y. Wang , L. Zhang , P. Wang , ACS Sustainable Chem. Eng. 2016, 4, 1223.

[68] L. Zhu , T. Ding , M. Gao , C. K. N. Peh , G. W. Ho , Adv. Energy Mater. 2019, 9, 1900250.

[69] V. D. Dao , H. S. Choi , Global Challenges 2018, 2, 1700094. 31565322 10.1002/gch2.201700094 PMC6607331

[70] Y. Bian , Q. Du , K. Tang , Y. Shen , L. Hao , D. Zhou , X. Wang , Z. Xu , H. Zhang , L. Zhao , S. Zhu , J. Ye , H. Lu , Y. Yang , R. Zhang , Y. Zheng , S. Gu , Adv. Mater. Technol. 2019, 4, 1800593.

[71] Z. Li , C. Wang , T. Lei , H. Ma , J. Su , S. Ling , W. Wang , Adv. Sustainable Syst. 2019, 3, 1800144.

[72] M. Zhu , Y. Li , G. Chen , F. Jiang , Z. Yang , X. Luo , Y. Wang , S. D. Lacey , J. Dai , C. Wang , C. Jia , J. Wan , Y. Yao , A. Gong , B. Yang , Z. Yu , S. Das , L. Hu , Adv. Mater. 2017, 29, 1704107. 10.1002/adma.201704107 29024077

[73] G. Xue , K. Liu , Q. Chen , P. Yang , J. Li , T. Ding , J. Duan , B. Qi , J. Zhou , ACS Appl. Mater. Interfaces 2017, 9, 15052. 28402107 10.1021/acsami.7b01992

[74] M. Zhu , J. Yu , C. Ma , C. Zhang , D. Wu , H. Zhu , Sol. Energy Mater. Sol. Cells 2019, 191, 83.

[75] Y. Zhang , S. K. Ravi , J. V. Vaghasiya , S. C. Tan , iScience 2018, 3, 31. 30428328 10.1016/j.isci.2018.04.003 PMC6137284

[76] Z. Li , C. Wang , Z. Li , L. Deng , J. Su , J. Shi , M. An , Energy Technol. 2019, 7, 1900406.

[77] J. Fang , J. Liu , J. Gu , Q. Liu , W. Zhang , H. Su , D. Zhang , Chem. Mater. 2018, 30, 6217.

[78] X. F. Luo , C. Ma , Z. Chen , X. Zhang , N. Niu , J. Li , S. Liu , S. Li , J. Mater. Chem. A 2019, 7, 4002.

[79] Y. Zhang , S. K. Ravi , S. C. Tan , Nano Energy 2019, 65, 104006.

[80] Z. Liu , H. Song , D. Ji , C. Li , A. Cheney , Y. Liu , N. Zhang , X. Zeng , B. Chen , J. Gao , Y. Li , X. Liu , D. Aga , S. Jiang , Z. Yu , Q. Gan , Global Challenges 2017, 1, 1600003. 28616256 10.1002/gch2.201600003 PMC5445597

[81] S. Wu , G. Xiong , H. Yang , Y. Tian , B. Gong , H. Wan , Y. Wang , T. S. Fisher , J. Yan , K. Cen , Z. Bo , K. Ostrikov , Matter 2019, 1, 1017.

[82] S. Wu , G. Xiong , H. Yang , B. Gong , Y. Tian , C. Xu , Y. Wang , T. Fisher , J. Yan , K. Cen , T. Luo , X. Tu , Z. Bo , K. Ostrikov , Adv. Energy Mater. 2019, 9, 1901286.

[83] X. Li , X. Min , J. Li , N. Xu , P. Zhu , B. Zhu , S. Zhu , J. Zhu , Joule 2018, 2, 2477.

[84] E. Chiavazzo , M. Morciano , F. Viglino , M. Fasano , P. Asinari , Nat. Sustain. 2018, 1, 763.

[85] Q. Shen , Z. Ning , B. Fu , S. Ma , Z. Wang , L. Shu , L. Zhang , X. Wang , J. Xu , P. Tao , C. Song , J. Wu , T. Deng , W. Shang , J. Mater. Chem. A 2019, 7, 6514.

[86] H. Liu , C. Chen , G. Chen , Y. Kuang , X. Zhao , J. Song , C. Jia , X. Xu , E. Hitz , H. Xie , S. Wang , F. Jiang , T. Li , Y. Li , A. Gong , R. Yang , S. Das , L. Hu , Adv. Energy Mater. 2018, 8, 1701616.

[87] C. Jia , Y. Li , Z. Yang , G. Chen , Y. Yao , F. Jiang , Y. Kuang , G. Pastel , H. Xie , B. Yang , S. Das , L. Hu , Joule 2017, 1, 588.

[88] C. Li , D. Jiang , B. Huo , M. Ding , C. Huang , D. Jia , H. Li , C. Liu , J. Liu , Nano Energy 2019, 60, 841.

[89] F. Wang , D. Wei , Y. Z. Li , T. Chen , P. Mu , H. Sun , Z. Zhu , W. Liang , A. Li , J. Mater. Chem. A 2019, 7, 18311.

[90] F. Jiang , H. Liu , Y. Li , Y. Kuang , X. Xu , C. Chen , H. Huang , C. Jia , X. Zhao , E. Hitz , Y. Zhou , R. Yang , L. Cui , L. Hu , ACS Appl. Mater. Interfaces 2018, 10, 1104. 29182304 10.1021/acsami.7b15125

[91] F. Ni , P. Xiao , C. Zhang , Y. Liang , J. C. Gu , L. Zhang , T. Chen , ACS Appl. Mater. Interfaces 2019, 11, 15498. 30964990 10.1021/acsami.9b00380

[92] Q. Fang , T. Li , H. Lin , R. Jiang , F. Liu , ACS Appl. Energy Mater. 2019, 2, 4354.

[93] H. Kou , Z. Liu , B. Zhu , D. K. Macharia , S. Ahmed , B. Wu , M. Zhu , X. Liu , Z. Chen , Desalination 2019, 462, 29.

[94] Q. Zhang , X. Xiao , G. Wang , X. Ming , X. Liu , H. Wang , H. Yang , W. Xu , X. Wang , J. Mater. Chem. A 2018, 6, 17212.

[95] F. Liu , L. Wang , R. Bradley , B. Zhao , W. Wu , RSC Adv. 2019, 9, 29414. 10.1039/c9ra05637h PMC9071831 35528431

[96] N. Xu , X. Hu , W. Xu , X. Li , L. Zhou , S. Zhu , J. Zhu , Adv. Mater. 2017, 29, 1606762. 10.1002/adma.201606762 28520092

[97] Y. Liu , Z. Liu , Q. Huang , X. Liang , X. Zhou , H. Fu , Q. Wu , J. Zhang , W. Xie , J. Mater. Chem. A 2019, 7, 2581.

[98] T. Gao , Y. Li , C. Chen , Z. Yang , Y. Kuang , C. Jia , J. Song , E. M. Hitz , B. Liu , H. Huang , J. Yu , B. Yang , L. Hu , Small Methods 2019, 3, 1800176.

[99] F. Liu , B. Zhao , W. Wu , H. Yang , Y. Ning , Y. Lai , R. Bradley , Adv. Funct. Mater. 2018, 28, 1803266.

[100] C. Guo , E. Miao , J. Zhao , L. Liang , Q. Liu , Sol. Energy 2019, 188, 1283.

[101] Y. Wang , C. Wang , X. Song , M. Huang , S. K. Megarajan , S. F. Shaukat , H. Jiang , J. Mater. Chem. A 2018, 6, 9874.

[102] C. Finnerty , L. Zhang , D. L. Sedlak , K. L. Nelson , B. Mi , Environ. Sci. Technol. 2017, 51, 11701. 28892371 10.1021/acs.est.7b03040

[103] X. Wu , L. Wu , J. Tan , G. Chen , G. Owens , H. Xu , J. Mater. Chem. A 2018, 6, 12267.

[104] X. Gao , H. Ren , J. Zhou , R. Du , C. Yin , R. Liu , H. Peng , L. Tong , Z. Liu , J. Zhang , Chem. Mater. 2017, 29, 5777.

[105] X. Wang , Q. Liu , S. Wu , B. Xu , H. Xu , Adv. Mater. 2019, 31, 1807716. 10.1002/adma.201807716 30920701

[106] W. Xu , Y. Xing , J. Liu , H. Wu , Y. Cui , D. Li , D. Guo , C. Li , A. Liu , H. Bai , ACS Nano 2019, 13, 7930. 31241310 10.1021/acsnano.9b02331

[107] K. Li , T.‐H. Chang , Z. Li , H. Yang , F. Fu , T. Li , J. S. Ho , P.‐Y. Chen , Adv. Energy Mater. 2019, 9, 1901687.

[108] X. Lin , J. Chen , Z. Yuan , M. Yang , G. Chen , D. Yu , M. Zhang , W. Hong , X. Chen , J. Mater. Chem. A 2018, 6, 4642.

[109] Z. Chen , B. Dang , X. Luo , W. Li , J. Li , H. Yu , S. Liu , S. Li , ACS Appl. Mater. Interfaces 2019, 11, 26032. 31259513 10.1021/acsami.9b08244

[110] Y. He , H. Li , X. Guo , R. Zheng , BioResources 2019, 14, 3758.

[111] C. Chen , Y. Li , J. Song , Z. Yang , Y. Kuang , E. Hitz , C. Jia , A. Gong , F. Jiang , J. Zhu , B. Yang , J. Xie , L. Hu , Adv. Mater. 2017, 29, 1701756. 10.1002/adma.201701756 28605077

[112] X. Wu , G. Y. Chen , W. Zhang , X. Liu , H. Xu , Adv. Sustainable Syst. 2017, 1, 1700046.

[113] Y. Tian , H. Yang , S. Wu , J. Yan , K. Cen , T. Luo , G. Xiong , Y. Hou , Z. Bo , K. Ostrikov , Nano Energy 2019, 66, 104125.

[114] Y. Shi , R. Li , Y. Jin , S. Zhuo , L. Shi , J. Chang , S. Hong , K. C. Ng , P. Wang , Joule 2018, 2, 1171.

[115] W. Li , Z. Li , K. Bertelsmann , D. Fan , Adv. Mater. 2019, 31, 1900720. 10.1002/adma.201900720 31134676

[116] S. Hong , Y. Shi , R. Li , C. Zhang , Y. Jin , P. Wang , ACS Appl. Mater. Interfaces 2018, 10, 28517. 30109921 10.1021/acsami.8b07150

[117] P. Zhang , Q. Liao , H. Yao , H. Cheng , Y. Huang , C. Yang , L. Jiang , L. Qu , J. Mater. Chem. A 2018, 6, 15303.

[118] X. Li , J. Li , J. Lu , N. Xu , C. Chen , X. Min , B. Zhu , H. Li , L. Zhou , S. Zhu , T. Zhang , J. Zhu , Joule 2018, 2, 1331.

[119] C. Tu , W. Cai , X. Chen , X. Ouyang , H. Zhang , Z. Zhang , Small 2019, 15, 1902070. 10.1002/smll.201902070 31379088

[120] Z. Yu , S. Cheng , C. Li , L. Li , J. Yang , ACS Appl. Mater. Interfaces 2019, 11, 32038. 31403274 10.1021/acsami.9b08480

[121] H. Song , Y. Liu , Z. Liu , M. H. Singer , C. Li , A. R. Cheney , D. Ji , L. Zhou , N. Zhang , X. Zeng , Z. Bei , Z. Yu , S. Jiang , Q. Gan , Adv. Sci. 2018, 5, 1800222. 10.1002/advs.201800222 PMC6096986 30128237

[122] X. Zhou , Y. Guo , F. Zhao , G. Yu , Acc. Chem. Res. 2019, 52, 3244. 31633912 10.1021/acs.accounts.9b00455

[123] F. Zhao , X. Zhou , Y. Shi , X. Qian , M. Alexander , X. Zhao , S. Mendez , R. Yang , L. Qu , G. Yu , Nat. Nanotechnol. 2018, 13, 489. 29610528 10.1038/s41565-018-0097-z

[124] X. Zhou , F. Zhao , Y. Guo , B. Rosenberger , G. Yu , Sci. Adv. 2019, 5, eaaw5484. 31259243 10.1126/sciadv.aaw5484 PMC6599166

[125] Y. Guo , F. Zhao , X. Zhou , Z. Chen , G. Yu , Nano Lett. 2019, 19, 2530. 30836007 10.1021/acs.nanolett.9b00252

[126] Y. Zhang , S. K. Ravi , L. Yang , J. V. Vaghasiya , L. Suresh , I. Tan , S. C. Tan , ACS Appl. Mater. Interfaces 2019, 11, 38674. 31560189 10.1021/acsami.9b12156

[127] H. Geng , Q. Xu , M. Wu , H. Ma , P. Zhang , T. Gao , L. Qu , T. Ma , C. Li , Nat. Commun. 2019, 10, 1512. 30944322 10.1038/s41467-019-09535-w PMC6447597

[128] T. Chen , S. Wang , Z. Wu , X. Wang , J. Peng , B. Wu , J. Cui , X. Fang , Y. Xiec , N. Zheng , J. Mater. Chem. A 2018, 6, 14571.

[129] B. Zhu , H. Kou , Z. Liu , Z. Wang , D. K. Macharia , M. Zhu , B. Wu , X. Liu , Z. Chen , ACS Appl. Mater. Interfaces 2019, 11, 35005. 31466452 10.1021/acsami.9b12806

[130] Y. Jin , J. Chang , Y. Shi , L. Shi , S. Hong , P. Wang , J. Mater. Chem. A 2018, 6, 7942.

[131] X. Feng , J. Zhao , D. Sun , L. Shanmugam , J. Kim , J. Yang , J. Mater. Chem. A 2019, 7, 4400.

[132] L. Shi , Y. Shi , R. Li , J. Chang , N. Zaouri , E. Ahmed , Y. Jin , C. Zhang , S. Zhuo , P. Wang , ACS Sustainable Chem. Eng. 2018, 6, 8192.

[133] H. Ren , M. Tang , B. Guan , K. Wang , J. Yang , F. Wang , M. Wang , J. Shan , Z. Chen , D. Wei , H. Peng , Z. Liu , Adv. Mater. 2017, 29, 1702590. 10.1002/adma.201702590 28833544

[134] Y. Kuang , C. Chen , S. He , E. Hitz , Y. Wang , W. Gan , R. Mi , L. Hu , Adv. Mater. 2019, 31, 1900498. 10.1002/adma.201900498 30989752

[135] S. He , C. Chen , Y. Kuang , R. Mi , Y. Liu , Y. Pei , W. Kong , W. Gan , H. Xie , E. Hitz , C. Jia , X. Chen , A. Gong , J. Liao , J. Li , Z. J. Ren , B. Yang , S. Das , L. Hu , Energy Environ. Sci. 2019, 12, 1558.

[136] G. Ni , S. H. Zandavi , S. M. Javid , S. V. Boriskina , T. A. Cooper , G. Chen , Energy Environ. Sci. 2018, 11, 1510.

[137] Q. Zhang , H. Yang , X. Xiao , H. Wang , L. Yan , Z. Shi , Y. Chen , W. Xu , X. Wang , J. Mater. Chem. A 2019, 7, 14620.

[138] L. Zhang , B. Tang , J. Wu , R. Li , P. Wang , Adv. Mater. 2015, 27, 4889. 26184454 10.1002/adma.201502362

[139] Y. Liu , J. Chen , D. Guo , M. Cao , L. Jiang , ACS Appl. Mater. Interfaces 2015, 7, 13645. 26027770 10.1021/acsami.5b03435

[140] Y. Yang , X. Yang , L. Fu , M. Zou , A. Cao , Y. Du , Q. Yuan , C. Yan , ACS Energy Lett. 2018, 3, 1165.

[141] W. Xu , X. Hu , S. Zhuang , Y. Wang , X. Li , L. Zhou , S. Zhu , J. Zhu , Adv. Energy Mater. 2018, 8, 1702884.

[142] R. Hu , J. Zhang , Y. Kuang , K. Wang , X. Cai , Z. Fang , W. Huang , G. Chen , Z. Wang , J. Mater. Chem. A 2019, 7, 15333.

[143] Y. Yang , H. Zhao , Z. Yin , J. Zhao , X. Yin , N. Li , D. Yin , Y. Li , B. Lei , Y. Du , W. Que , Mater. Horiz. 2018, 5, 1143.

[144] N. Xu , J. Li , Y. Wang , C. Fang , X. Li , Y. Wang , L. Zhou , B. Zhu , Z. Wu , S. Zhu , J. Zhu , Sci. Adv. 2019, 5, eaaw7013. 31281896 10.1126/sciadv.aaw7013 PMC6611683

[145] Y. Xia , Q. Hou , H. Jubaer , Y. Li , Y. Kang , S. Yuan , H. Y. Liu , M. W. Woo , L. Zhang , L. Gao , H. Wang , X. Zhang , Energy Environ. Sci. 2019, 12, 1840.

[146] Y. Shi , C. Zhang , R. Li , S. Zhuo , Y. Jin , L. Shi , S. Hong , J. Chang , C. S. Ong , P. Wang , Environ. Sci. Technol. 2018, 52, 11822. 30221518 10.1021/acs.est.8b03300

[147] Z. Liu , B. Wu , B. Zhu , Z. Chen , M. Zhu , X. Liu , Adv. Funct. Mater. 2019, 29, 1905485.

[148] X. Li , G. Ni , T. Cooper , N. Xu , J. Li , L. Zhou , X. Hu , B. Zhu , P. Yao , J. Zhu , Joule 2019, 3, 1798.

[149] X. Wu , M. E. Robson , J. L. Phelps , J. Tan , B. Shao , G. Owens , H. Xu , Nano Energy 2019, 56, 708.

[150] C. Chang , P. Tao , J. Xu , B. Fu , C. Song , J. Wu , W. Shang , T. Deng , ACS Appl. Mater. Interfaces 2019, 11, 18466. 31046219 10.1021/acsami.9b04535

[151] J. Li , M. Du , G. Lv , L. Zhou , X. Li , L. Bertoluzzi , C. Liu , S. Zhu , J. Zhu , Adv. Mater. 2018, 30, 1805159. 10.1002/adma.201805159 30303571

[152] Y. Zhang , D. Zhao , F. Yu , C. Yang , J. Lou , Y. Liu , Y. Chen , Z. Wang , P. Tao , W. Shang , J. Wu , C. Song , T. Deng , Nanoscale 2017, 9, 19384. 29206253 10.1039/c7nr06861a

[153] P. Yang , K. Liu , Q. Chen , J. Li , J. Duan , G. Xue , Z. Xu , W. Xie , J. Zhou , Energy Environ. Sci. 2017, 10, 1923.

[154] Z. Zhang , X. Li , J. Yin , Y. Xu , W. Fei , M. Xue , Q. Wang , J. Zhou , W. Guo , Nat. Nanotechnol. 2018, 13, 1109. 30523296 10.1038/s41565-018-0228-6

[155] C. Li , Z. Tian , L. Liang , S. Yin , P. K. Shen , ACS Appl. Mater. Interfaces 2019, 11, 4922. 30632734 10.1021/acsami.8b16529

[156] F. Yi , H. Ren , K. Dai , X. Wang , Y. Han , K. Wang , K. Li , B. Guan , J. Wang , M. Tang , J. Shan , H. Yang , M. Zheng , Z. You , D. Wei , Z. Liu , Energy Environ. Sci. 2018, 11, 2016.

[157] L. Yang , S. K. Ravi , D. K. Nandakumar , F. I. Alzakia , W. Lu , Y. Zhang , J. Yang , Q. Zhang , X. Zhang , S. C. Tan , Adv. Mater. 2019, 31, 1902963. 10.1002/adma.201902963 31650636

[158] D. K. Nandakumar , S. K. Ravi , Y. Zhang , N. Guo , C. Zhang , S. C. Tan , Energy Environ. Sci. 2019, 11, 2179.

[159] W. Wang , Y. Shi , C. Zhang , S. Hong , L. Shi , J. Chang , R. Li , Y. Jin , C. Ong , S. Zhuo , P. Wang , Nat. Commun. 2019, 10, 3012. 31289262 10.1038/s41467-019-10817-6 PMC6616361

[160] Z. Xu , L. Zhang , L. Zhao , B. Li , B. Bhatia , C. Wang , K. L. Wilke , Y. Song , O. Labban , J. H. Lienhard , R. Z. Wang , E. Wang , Energy Environ. Sci. 2020, 10.1039/C9EE04122B.

[161] X. Zha , X. Zhao , J. Pu , L. Tang , K. Ke , R. Bao , L. Bai , Z. Liu , M. Yang , W. Yang , ACS Appl. Mater. Interfaces 2019, 11, 36589. 31513743 10.1021/acsami.9b10606

[162] X.‐Y. Wang , J. Xue , C. Ma , T. He , H. Qian , B. Wang , J. Liu , Y. Lu , J. Mater. Chem. A 2019, 7, 16696.

